# Cross-species blood transcriptional correlates of BCG-mediated protection against tuberculosis include innate and adaptive immune processes

**DOI:** 10.1172/jci.insight.194450

**Published:** 2025-11-11

**Authors:** Kate Bridges, Denis Awany, Anele Gela, Temwa-Dango Mwambene, Sherry L. Kurtz, Richard E. Baker, Karen L. Elkins, Christopher M. Sassetti, Thomas J. Scriba, Douglas A. Lauffenburger

**Affiliations:** 1 Department of Biological Engineering, Massachusetts Institute of Technology, Cambridge, Massachusetts, USA.; 2 South African Tuberculosis Vaccine Initiative, Institute of Infectious Disease and Molecular Medicine and Division of Immunology, Department of Pathology, University of Cape Town, Cape Town, South Africa.; 3 Center for Biologics Evaluation and Research, Food and Drug Administration, Silver Spring, Maryland, USA.; 4 Department of Microbiology and Physiological Systems, UMass Chan Medical School, Worcester, Massachusetts, USA.

**Keywords:** Immunology, Infectious disease, Bacterial vaccines, Cellular immune response, Tuberculosis

## Abstract

The immune mechanisms induced by the Bacillus Calmette-Guérin (BCG) vaccine, and the subset of which that mediate protection against tuberculosis (TB), remain poorly understood. This is further complicated by difficulties in verifying vaccine-induced protection in humans. Although research in animal models, namely mice and nonhuman primates (NHPs), has begun to close this knowledge gap, discrepancies in the relative importance of biological pathways across species limit the utility of animal model–derived biological insights in humans. To address these challenges, we applied a systems modeling framework, Translatable Components Regression (TransCompR), to identify human blood transcriptional variability that could predict *Mycobacterium tuberculosis* challenge outcomes in BCG-vaccinated NHPs. These protection-associated pathways included both innate and adaptive immune activation mechanisms, along with signaling via type I IFNs and antimycobacterial Th cytokines. We further partially validated the associations between these mechanisms and protection in humans using publicly available microarray data collected from BCG-vaccinated infants who either developed TB or remained healthy during 2 years of follow-up. Overall, our work demonstrates how species translation modeling can leverage animal studies to generate hypotheses about the mechanisms that underlie human infectious disease and vaccination outcomes, which may be difficult or impossible to ascertain using human data alone.

## Abstract 2

By translating insights from animal models, our work overcomes limitations in clinical data to identify human immune mechanisms associated with successful vaccine outcomes.

## Introduction

Tuberculosis (TB) is presently the deadliest infectious disease caused by a single agent, with 10.8 million incident cases and 1.25 million deaths globally in 2023 (1). Among preventive interventions, the only TB vaccine available is the Bacillus Calmette-Guérin (BCG) vaccine (2), which is typically administered intradermally at birth in high-incidence countries. Despite efficacy against disseminated disease in young children, BCG confers variable protection against adolescent and adult pulmonary TB (3–5) via mechanisms that remain poorly understood by the field. Robust vaccine-mediated immune correlates of protection need to be identified to better inform the development and use of TB vaccines.

A major challenge with TB vaccine research is the difficulty in assessing vaccine-mediated protection in humans. At minimum, studying postvaccination clinical outcomes in humans requires clinical follow-up of thousands of participants (6–8) where exposure to pathogen is not always a known variable. Study designs also may not include an unvaccinated control group because it would violate the bioethical principles of beneficence and nonmaleficence. Moreover, diagnostic tools for TB vary widely in their sensitivity, specificity, cost, portability, and resource intensity (9–11), which complicate their application to large human cohorts in different ways and limit disease classification. Some clinical outcomes, such as bacterial burden in lung tissues, are simply not directly observable in living humans given present diagnostic tools and ethical considerations (12–14).

For these reasons, TB vaccine research heavily relies on the use of animal models, including mice and nonhuman primates (NHPs), to evaluate vaccine-mediated immune mechanisms (15, 16). However, animal model–derived biological insights are not guaranteed to be directly translatable to humans (17, 18), owing in part to divergent immune processes (19, 20) and to discrepancies in the phenotypic relevance of conserved immune processes (21) across species. For example, the long-held paradigm regarding the mechanism of action of BCG was that protective immunity is conferred by vaccine-induced IFN-γ–producing CD4^+^ T cells (22), which was supported in part by mouse studies (23, 24). However, various clinical studies have asserted little to no correlation between IFN-γ^+^CD4^+^ T cells and BCG-mediated protection (6, 25), suggesting that cross-species differences could be one factor that limits the translational potential of TB vaccine studies in animal models. This example in particular highlights the need for the development and widespread use of improved approaches to enhance translation proficiency (26–28).

Our group previously developed a computational framework called Translatable Components Regression (TransCompR), which was designed to relate orthogonal axes of transcriptional or proteomic variability in one species to disease biology and measurable phenotypes observed in another species (27). In doing so, TransCompR explicitly accounts for differences in the relative importance of disease-relevant processes and pathways in the two species. Although originally applied in the contexts of inflammatory bowel disease (27, 29) and neuropathology (30, 31), we have recently used this framework to identify transcriptional variability in mouse TB models that could best predict human TB phenotypes (21, 32), demonstrating the utility of TransCompR to uncover species-translatable signatures in an infectious disease context.

In this work, we adapted TransCompR to identify human-relevant biological pathways that are associated with the outcome of post–*M*. *tuberculosis* challenge in BCG-vaccinated NHPs. We show that a linear mathematical model built on human blood transcriptomic data could predict vaccine-mediated protection against TB, as assessed in publicly available NHP studies (33, 34). These protection-associated directions of human transcriptional variance corresponded to the activation of innate and adaptive immune mechanisms, including type I IFN and antimycobacterial Th cytokine signaling. We further partially validated the associations between these directions of transcriptional variance and protection using publicly available microarray data from a heterogeneous cohort of BCG-vaccinated infants who either developed TB or remained healthy during 2 years of follow-up (35). Overall, we demonstrate how translational cross-species modeling can leverage animal studies to uncover mechanisms of action in humans where no outcome data are available.

## Results

### Bulk blood transcriptomics data collection characterizes BCG-induced biological variability across species.

**Figure 1:**
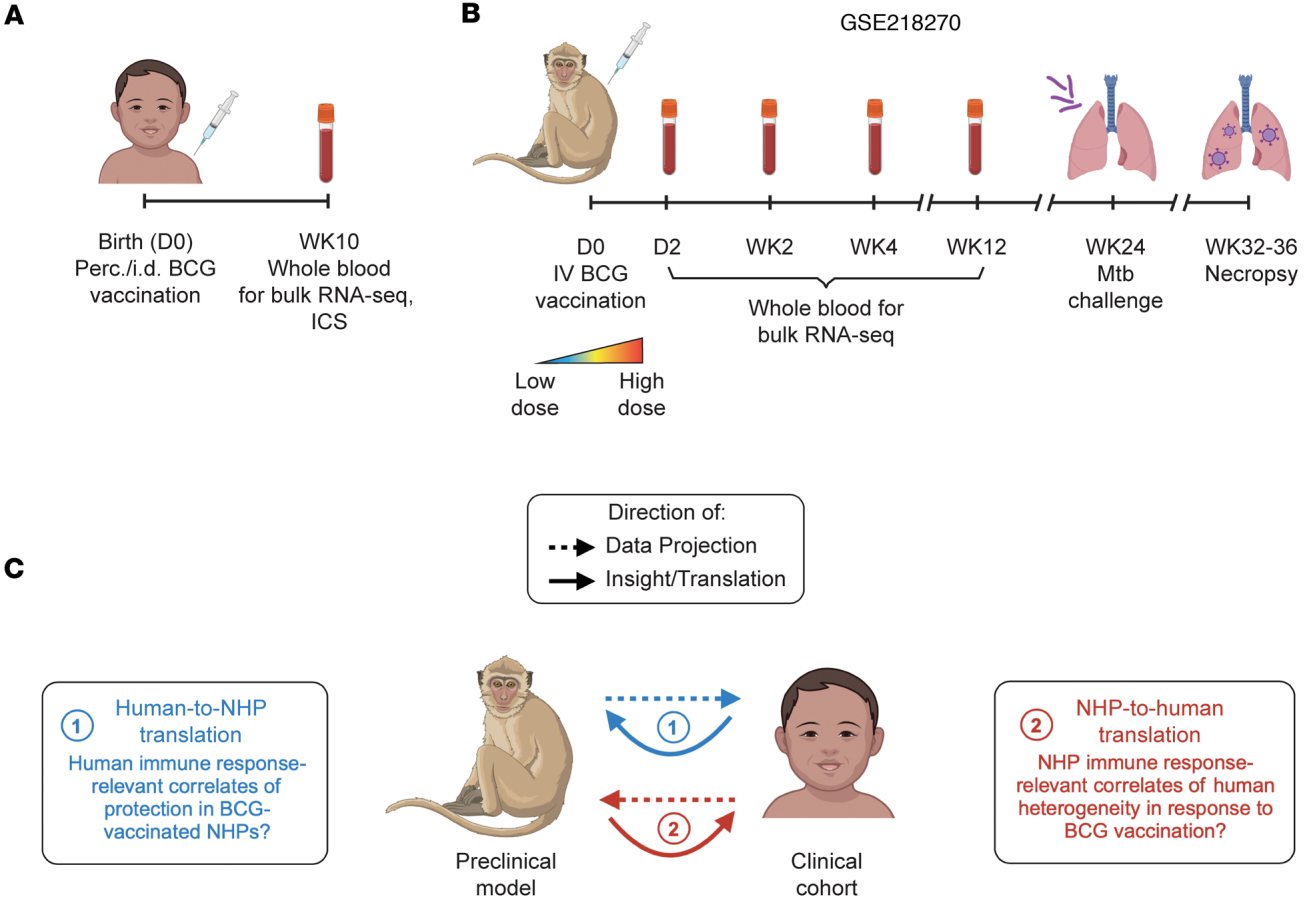
BCG-induced patterns in gene expression across species. (**A**) Schematic showing collection of blood for bulk RNA-seq and profiling with intracellular cytokine staining (ICS) in a cohort of 60 BCG-vaccinated South African infants. (**B**) Schematic showing collection of blood for bulk RNA-seq in a cohort of 34 IV BCG-vaccinated nonhuman primates (NHPs). Vaccination was administered over a range of BCG doses. (**C**) Schematic showing species translation conceptually. Translation modeling involving two species presents two potential directions for analysis, which affects the biological questions that can be probed computationally.

To develop an understanding of vaccine-induced immune responses in animal models and humans, we used NHP and human bulk blood transcriptomic data. We included data from 60 South African infants who received percutaneous or intradermal BCG vaccination at birth and were selected from a parent cohort study of 11,680 infants (6, 7) to reflect a range of BCG responses based on antigen-induced T cell IFN-γ production (see Methods). Bulk transcriptional profiling on peripheral blood mononuclear cells (PBMCs) and intracellular cytokine staining (ICS) on whole blood collected at 10 weeks of age were performed for characterization of peripheral immune responses (Figure 1A, Supplemental Figure 1A, and Supplemental Table 1; supplemental material available online with this article; https://doi.org/10.1172/jci.insight.194450DS1). The NHP dataset was previously published in a study by Liu et al. (33). NHPs in this cohort (*n* = 34; “dose cohort”; ref. 36) were vaccinated intravenously with BCG across a range of doses, and whole blood was collected for transcriptional profiling at baseline and at 4 postvaccination time points (Figure 1B and Supplemental Table 2). Plasma and bronchoalveolar lavage (BAL) samples were also collected at these or similar time points for flow cytometry and antibody profiling (37). At 24 weeks after vaccination, NHPs were challenged with *M*. *tuberculosis* and outcome was assessed at 8-12 weeks post–*M*. *tuberculosis* challenge via quantification of bacterial burden (as colony forming units [CFU]) in lung tissues.

### TransCompR model identifies human transcriptional variability associated with BCG-mediated protection in NHPs.

To construct a species translation model, we used TransCompR (27), a method previously developed in our group to relate axes of data-derived variance in one species to a phenotype of interest in another species. Like other species translation approaches, TransCompR analyses have directionality (Figure 1C). Historically, TransCompR has been used to relate transcriptional variability in mouse or NHP models of disease to human disease characteristics (e.g., animal model–to-human translation, refs. 21, 27, 38; visualized in Figure 1C, in red) with the objective being to better understand axes of variance in animal models with respect to their human relevance. However, human-to-animal model translation (visualized in Figure 1C, in blue), in which human variance is regressed against biological outcomes as assessed in animal models, also offers actionable insights. This direction of species translation is particularly useful for studies in which assessing individuals’ responses to immunomodulation is difficult or limited by experimental or ethical bounds, a point that our group has previously demonstrated in noninfectious disease contexts (29, 30). Different directions of translation address different scientific questions; therefore, depending on the application, one of the two possible directions might address a more informative question than the other.

**Figure 2:**
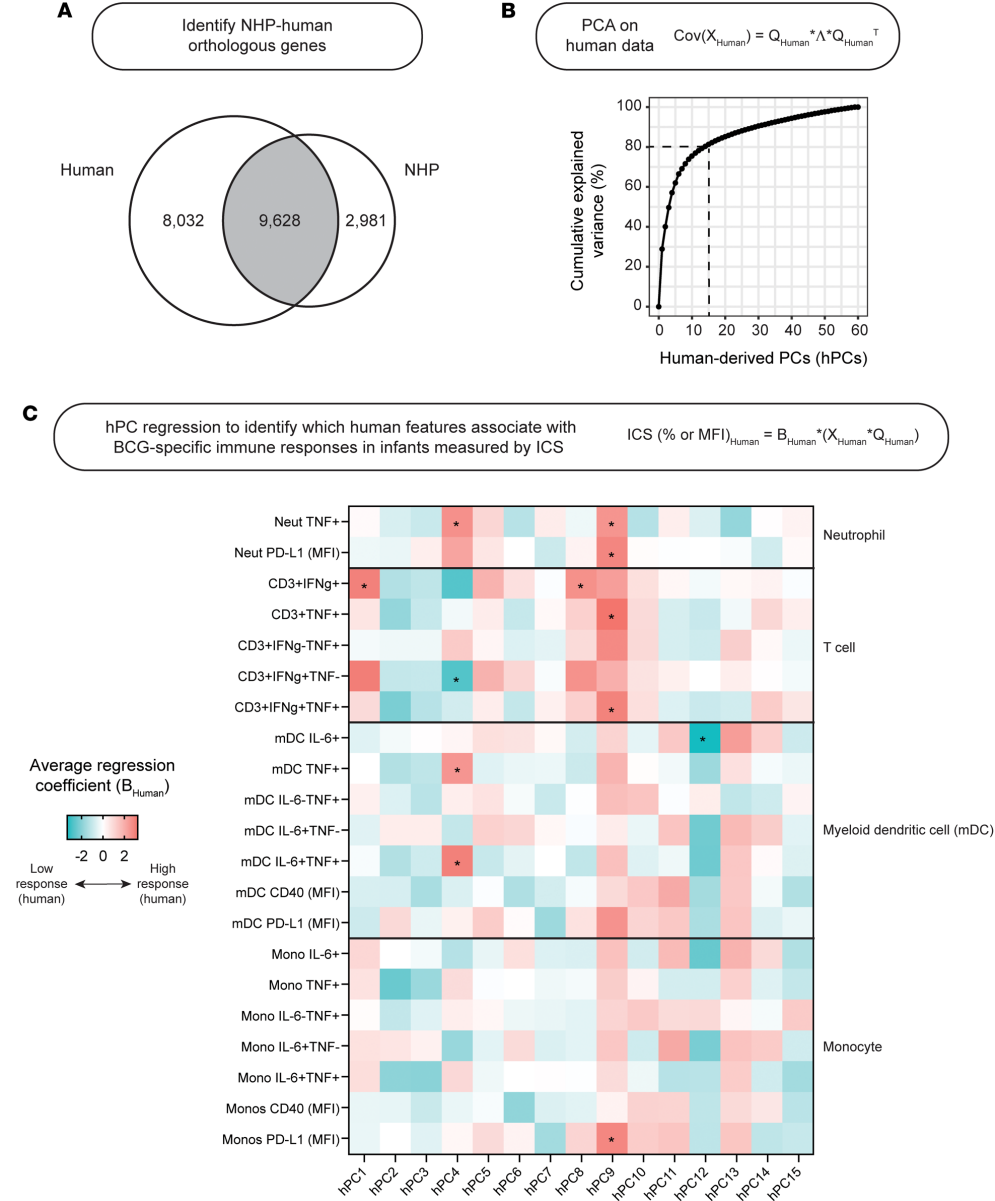
Human-derived principal components associate with BCG-specific immune responses in infants. (**A**) Venn diagram demonstrating the number of genes profiled in the human and NHP bulk RNA-seq datasets that were either species-specific or orthologous. (**B**) Line plot demonstrating cumulative explained variance (%) across subsequent human-derived principal components (hPCs) calculated from the human bulk RNA-seq dataset (limited to orthologous genes only). (**C**) Heatmap of univariate regression coefficients from individual hPCs regressed against patterns in cytokine expression (% unless otherwise indicated) from immune cells stimulated with BCG ex vivo. Regression coefficients were averaged across results from 5-fold cross-validation. *P < 0.05 across folds. For each fold, *P* values were calculated by Wald’s test.

Because vaccine-induced protection is challenging to ascertain in humans, in this work we chose to pursue human-to-NHP translation modeling (Figure 1C, blue only) to investigate human immune response–relevant correlates of protection in BCG-vaccinated NHPs. To do this, we first identified 9,628 gene homologs between the human and NHP datasets for inclusion in our cross-species model (Figure 2A). We then used principal component analysis (PCA) on the human bulk RNA-seq data to construct a latent space which describes orthogonal directions of transcriptional variance in the South African infant cohort. The first 15 human-derived principal components (hPCs), which captured approximately 80% of total transcriptional variance in the infant cohort (Figure 2B), were retained for cross-species modeling.

Prior to translation, we were first interested to understand how these hPCs, representative of blood transcriptional variance, related to measurable human phenotypes. To do this, we univariately regressed each of the 15 hPCs retained in our model against patterns in cytokine expression by T cells, monocytes, myeloid dendritic cells (mDCs), or neutrophils, assessed by ICS on whole blood and collected at 10 weeks post-BCG priming; these cells were either left unstimulated or stimulated ex vivo with BCG or staphylococcal enterotoxin B (SEB), a positive control (Figure 2C and Supplemental Figure 1, A and B). Associations quantified by regression were notably different between SEB- and BCG-stimulated cells. We further found 5 of these 15 hPCs to be significantly associated with one or more BCG-specific cytokine-expressing cell subsets. hPC1, hPC4, and hPC8 were significantly associated with BCG-specific IFN-γ–expressing T cells, while hPC9 was instead significantly associated with BCG-specific TNF-expressing T cells. Interestingly, hPC12 was associated with IL-6–expressing mDCs, suggestive of human transcriptional variance that might correlate with innate responses to BCG. Because antigen-specific IFN-γ production by T cells is typically considered a protective immune marker in the context of BCG vaccination (22), we binned infants into high and low responder groups by their BCG-specific IFN-γ^+^ T cell response (Supplemental Figure 2A) and used univariate logistic regression to identify hPCs that were predictive of responder group. Separation between responder groups was not observed along the first 2 hPCs (Supplemental Figure 2B). Instead, hPC4, hPC8, and hPC9, which collectively explained 12% of total transcriptional variance, were found to significantly distinguish infants by responder group (Supplemental Figure 2, C and D). Using covariate logistic regression, these 3 hPCs were capable of predicting high versus low BCG-specific IFN-γ^+^ T cell response with high classification accuracy (Supplemental Figure 2E). This result signaled that intermediate- and higher-order hPCs (i.e., those that explain *less* transcriptional variance overall compared with low-order hPCs, such as hPC1 and hPC2) remain relevant to human immunological responses of interest.

**Figure 3:**
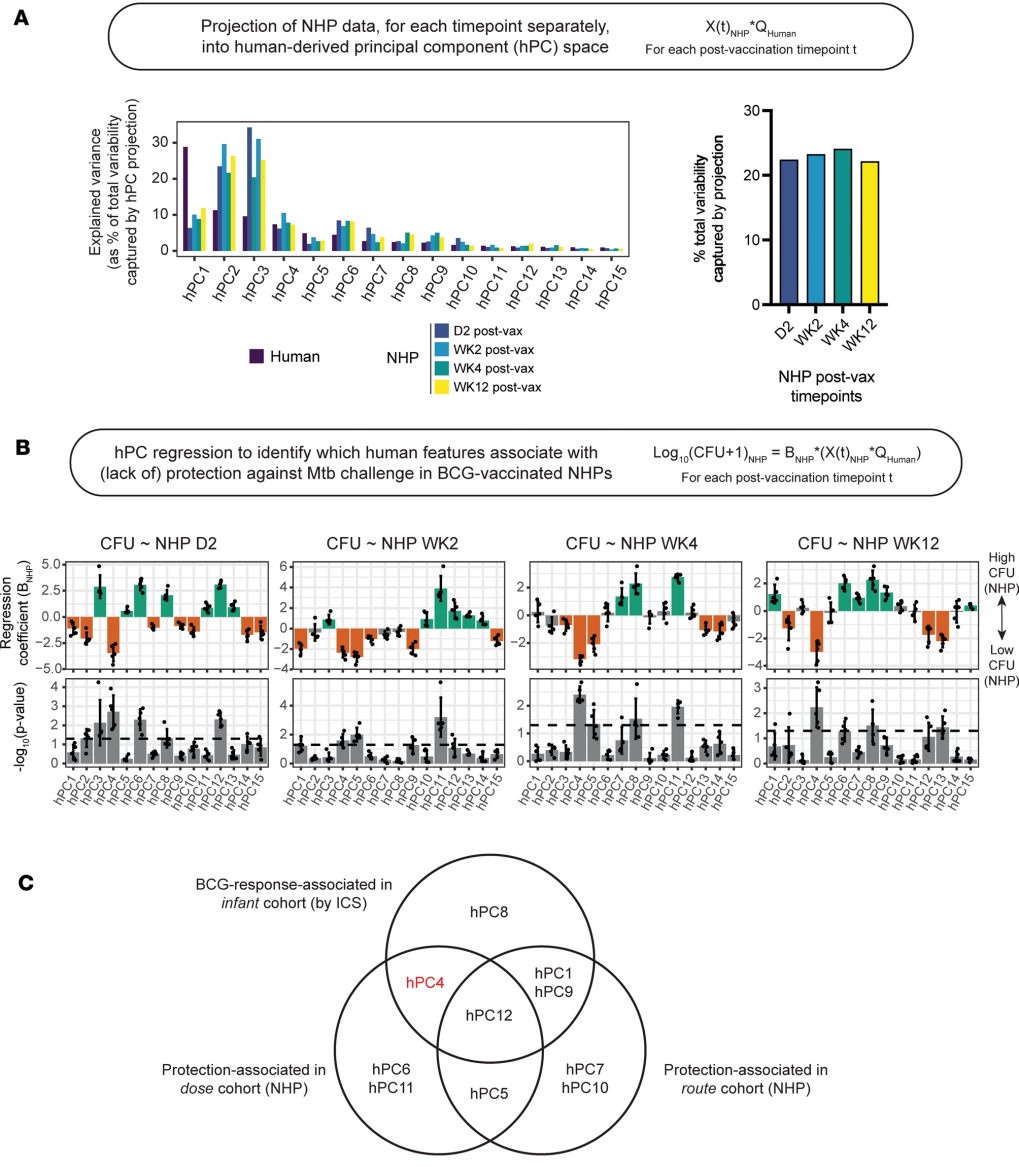
Human-derived principal components can predict post–*M*. *tuberculosis* challenge bacterial burden in NHPs. (**A**) Bar graphs showing explained variance captured by projecting the NHP bulk RNA-seq data, restricted to orthologous genes, into hPC space (right) as a percentage of total variance preserved for each postvaccination NHP dataset and (left) as distributed across each hPC in the TransCompR model. (**B**) Bar graphs showing (top) 5-fold logistic regression coefficients (summary data presented as mean ± SD) and (bottom) distribution of corresponding *P* values from univariate regression of each hPC against post–*M*. *tuberculosis* challenge bacterial burden (CFU). For each fold, *P* values are calculated by Wald’s test. The dotted line denotes *P* = 0.05. (**C**) Venn diagram detailing overlap of hPCs, which were found to be significantly associated with protection in the dose (**B**) and route (Supplemental Figure 5B) cohorts of NHPs and with BCG-specific immune cytokine expression in the South African infant cohort (Figure 2C). hPC4 is highlighted in red to indicate its unexpected correspondence between high bacterial burden in NHPs and high BCG-specific T cell response in infants.

To next approach species translation, we projected each postvaccination NHP sample into hPC space using the gene ortholog loadings for each of the 15 hPCs retained in our model, resulting in NHP sample scores along these hPC axes. This projection allowed us to “reorder” or “filter” the NHP data to reflect the most relevant directions of variance in the human data. As expected, for human samples, the variance explained by each subsequent hPC monotonically decreased, while this trend was broken for projected NHP samples (Figure 3A). Interestingly, while patterns in explained variability were similar for each NHP time point, they were not identical, which hinted at how axes of variance in the data might change over time. Moreover, across postvaccination time points, hPC projection preserved approximately 25% of the total variance in the NHP datasets (Figure 3A), which reemphasized the idea that common biological pathways and processes may differentially contribute to transcriptional variability, and, ultimately, higher order biological function, across species.

Because many of the immune responses quantified in the dose cohort of NHPs exhibited dose dependence (4), we were first interested to understand how dose-linked transcriptional variance might be reflected in the hPC-projected bulk RNA-seq data. To do this, we univariately regressed the hPC scores from the projected NHP samples against the dose of vaccination administered to each NHP. Many hPCs were significantly associated with BCG dose (Supplemental Figure 3), both with and without time dependence, suggesting that dose-linked variation was reflected at the transcriptional level and that this variation was preserved upon hPC projection. This preservation also raised the possibility that the range of immune responses generated in NHPs by varying BCG dose maintained some human relevance. hPC4 was also significantly associated with BCG dose in NHPs at all postvaccination time points, suggesting that this component captures variance related to robust and sustained response mechanisms that are potentially core and conserved in both species.

Finally, toward our goal of identifying vaccine-induced correlates of protection in the NHP data that are human-relevant, we then, for each postvaccination time point separately, univariately regressed hPC scores from the projected NHP samples against post–*M*. *tuberculosis* challenge bacterial burden to test for statistically significant association (Figure 3B). This analysis identified 5 protection-associated hPCs (with time dependence): hPC4 (all postvaccination time points); hPC5 (2 weeks); hPC6 (2 days); hPC11 (2 weeks and 4 weeks); and hPC12 (2 days). These associations largely held whether post–*M*. *tuberculosis* challenge outcome was treated as a continuous (CFU) or discrete (“protected” versus “not protected”) variable, and covariate regression using each time-specific subset of protection-associated hPCs confirmed significant improvement in prediction of postchallenge outcome compared with null models with random hPCs or shuffled outcomes (Supplemental Figure 4).

To examine the robustness of associations between hPCs in our model and protection against TB, we repeated the projection and regression steps of our species translation approach using an independent cohort of NHPs (Supplemental Figure 5, A and B). Referred to as the “route cohort” (33, 34), these NHPs (*n* = 34) were vaccinated with BCG via different routes, and, similarly to the dose cohort, blood was collected for transcriptional profiling at baseline and at 3 postvaccination time points, with later *M*. *tuberculosis* challenge and necropsy to assess protection against disease. Like the dose cohort, hPC projection preserved approximately 20%–25% of the total variance in the NHP route cohort data (Supplemental Figure 5A). Regressing hPC-projected route cohort samples against post–*M*. *tuberculosis* challenge bacterial burden provided further support for hPC5 (2 week) and hPC12 (2 days) as time-dependent, species-translatable axes of variance that were associated with protection against TB across independent NHP cohorts (Supplemental Figure 5, B and C).

Finally, we examined the overlap between hPCs significantly associated with protection across NHP cohorts and hPCs significantly associated with BCG-specific immune responses in infants (Figure 3C and Supplemental Figure 5D). Perhaps expectedly, 3 T cell response-associated hPCs (hPC1, hPC4, and hPC9) were also significantly associated with protection in 1 of the 2 NHP cohorts, although, contradictorily, for hPC4, protected NHP samples were separated on the same end of the axis as the low T cell responders (Supplemental Figure 4A; compare to Supplemental Figure 2D). Of the hPCs found to be protection-associated in both NHP cohorts, hPC5 did not correspond to any patterns in human immune cytokine expression that were assessed by ICS, while hPC12 was instead associated with antigen-reactive innate responses in infants (Figure 2C). Overall, our cross-species modeling identified axes of transcriptional variability that maintained significant associations with measurable human and NHP biological outcomes.

### Cross-species correlates of protection represent biological axes related to innate, adaptive, and humoral immunity.

We next wanted to biologically interpret these species-translatable hPCs, as they represent orthogonal directions of variance, and therefore we expected them to reflect distinct (although not wholly disjoint) biological processes. We focused on hPC5 and hPC12, which were protection-associated across both NHP cohorts, for these analyses. To manage this interpretation, we used gene set enrichment analysis (GSEA) to compare the gene loadings for these hPCs against gene sets in the Reactome Pathways and Kyoto Encyclopedia of Genes and Genomes (KEGG) databases (39, 40).

**Figure 4:**
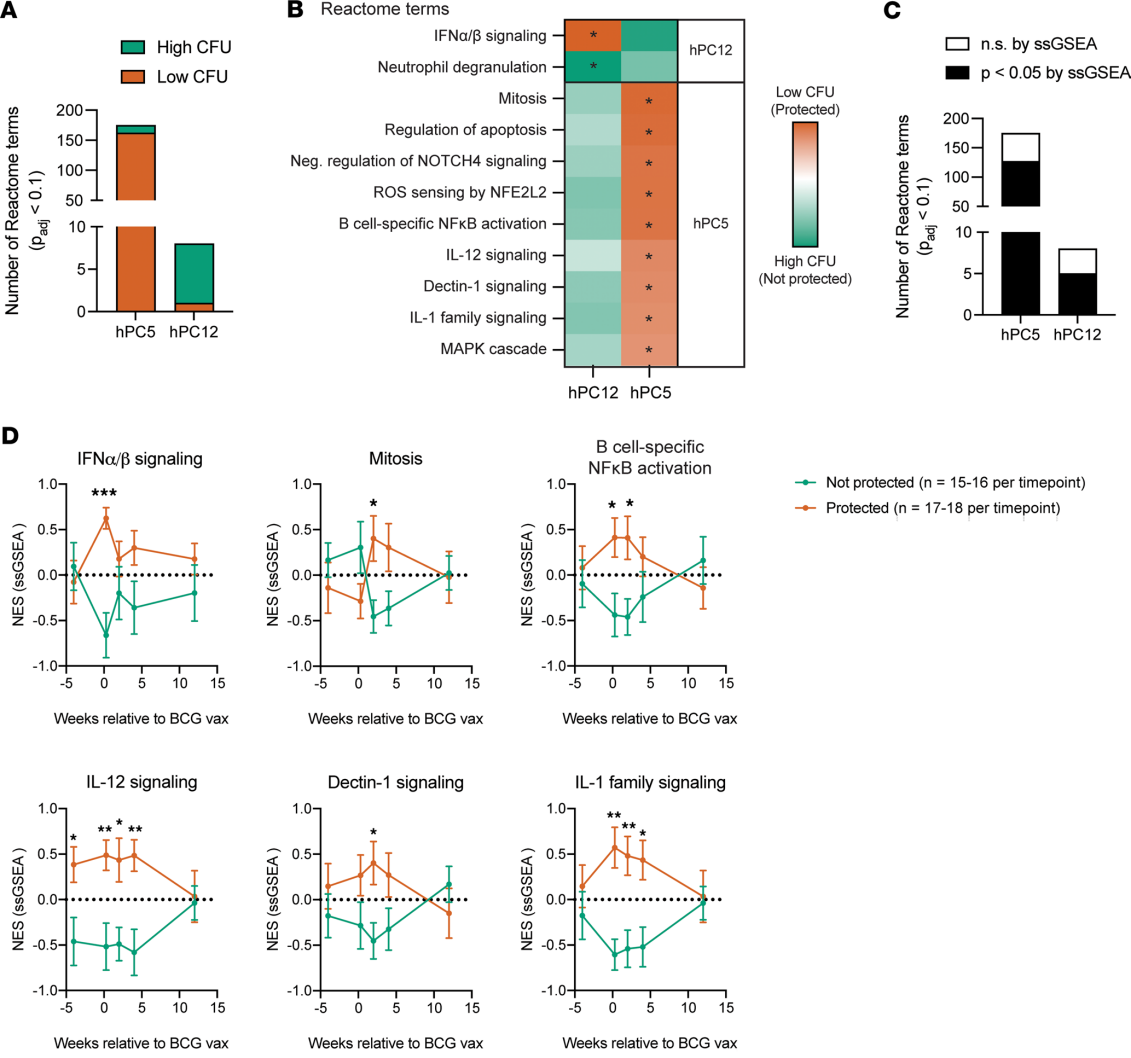
Protection-associated hPCs are enriched for innate and adaptive immune processes and pathways. (**A**) Bar graph demonstrating the total number of significantly enriched (*P*_adj_ < 0.1) Reactome terms along hPC5 and hPC12. (**B**) Heatmap showing enrichment scores for select Reactome terms across hPC5 and hPC12. *P_adj_ < 0.1. *P* values were estimated using an adaptive multilevel split Monte-Carlo scheme implemented in the fgsea package in R. (**C**) Bar graph as in **A**, with terms now separated into significant (black) or nonsignificant (white) categories by how their leading-edge gene sets distinguished NHP samples by challenge outcome (*P* values obtained by unpaired *t* test). hPC12-enriched terms were evaluated for their ability to separate NHP samples collected 2 days after vaccination by their challenge outcome, while hPC5-enriched terms were evaluated using NHP samples collected 2 weeks after vaccination. (**D**) Normalized enrichment scores (NESs) calculated using single-sample gene set enrichment analysis (ssGSEA) for each sample in the NHP dose cohort. Gene sets for each pathway/process were derived from the Reactome Pathway Database. Sample scores were lumped by post–*M*. *tuberculosis* challenge protection outcome for visualization. Data are presented as mean ± SEM. *P < 0.05, **P < 0.01, ***P < 0.001 by unpaired *t* test with Holm-Šídák’s multiple comparison correction, as calculated in GraphPad.

Using Reactome as the reference database, hPC5 (2 week) was significantly enriched (*P*_adj_ < 0.1) for 175 terms, while hPC12 (2 days) was significantly enriched for only 8 terms (Figure 4A and Supplemental Table 3). With KEGG, hPC5 and hPC12 were significantly enriched for 35 and 8 immune- and cell function-related terms, respectively (Supplemental Figure 6). Across reference databases, we noted enrichment of hPC12 for innate immune activation, antiviral immunity, and type I IFN signaling, mechanisms that have been shown to be altered by BCG, *M*. *tuberculosis* (41), and other pulmonary infections (42), while hPC5 was enriched for signaling pathways related to innate-adaptive immune crosstalk, Th differentiation, and B cell activation (Figure 4B and Supplemental Figure 6). Included in these terms was Dectin-1 signaling, enriched along hPC5. Dectin-1 is a C-type lectin that functions as a pattern recognition receptor, is primarily expressed on antigen-presenting cells (APCs) (43), including dendritic cells (DCs) and B cells, and has been described to play a role in immune responses to mycobacteria, including *M*. *tuberculosis* and BCG (44, 45). Signaling downstream of Dectin-1 activation is known to induce production of reactive oxygen species in neutrophils, as well as NF-κB– and MAPK-mediated expression of proinflammatory cytokines, including TNF, IL-12, IL-6, and IL-1β, in APCs (43, 46). Because IL-12 and IL-6/IL-1β are known to direct the differentiation of naive CD4^+^ T cells into Th subsets Th1 and Th17, respectively (47), it is possible that hPC5 represents an axis of transcriptional variability that is associated with Dectin-1–mediated coupling of innate and adaptive immunity.

To confirm that these hPC-enriched Reactome pathways and processes separated NHPs by their post–*M*. *tuberculosis* challenge infection status, we used single-sample GSEA (ssGSEA) to calculate enrichment scores for these gene sets for each individual sample in the NHP dose cohort at each profiled time point and compared between “protected” and “nonprotected” NHPs. As expected, we observed statistically significant, time-dependent separation of samples by the majority of hPC12- and hPC5-enriched terms (Figure 4C), including the IFN and cytokine signaling pathways that we highlighted (Figure 4D), at one or more postvaccination time points. Repeating this analysis in the route cohort of NHPs, we observed similar patterns in time-dependent enrichment, which distinguished protected from nonprotected NHPs (Supplemental Figure 7A).

Although NHPs are important animal models for studying TB, owing to many genetic and immunological similarities to humans, their research applications can be limited by low availability and high maintenance costs. Because mouse models are often used in their stead, we were interested to see whether these hPC-enriched Reactome pathways and processes were further predictive of post–*M*. *tuberculosis* challenge lung bacterial burden in mice. To test this, we took advantage of a blood bulk RNA-seq dataset that captured Diversity Outbred (DO) mice, a heterogeneous stock derived from 8 founder inbred strains (48), before and 2–3 weeks after BCG vaccination. We then calculated enrichment scores with ssGSEA directly on the dataset as before. On average, we observed increased enrichment after vaccination for many of the selected Reactome pathways and processes from samples from protected versus nonprotected mice, although we could only assert a statistically significant increase in enrichment for Dectin-1 signaling (Supplemental Figure 7B). Overall, interpreting protection-associated hPCs with gene set enrichment analyses identified species-translatable biological pathways and processes that were associated with BCG-mediated protection in NHPs.

### Independent cohort of South African infants with 2-year follow-up outcomes partially validates species-translatable correlates of protection.

**Figure 5:**
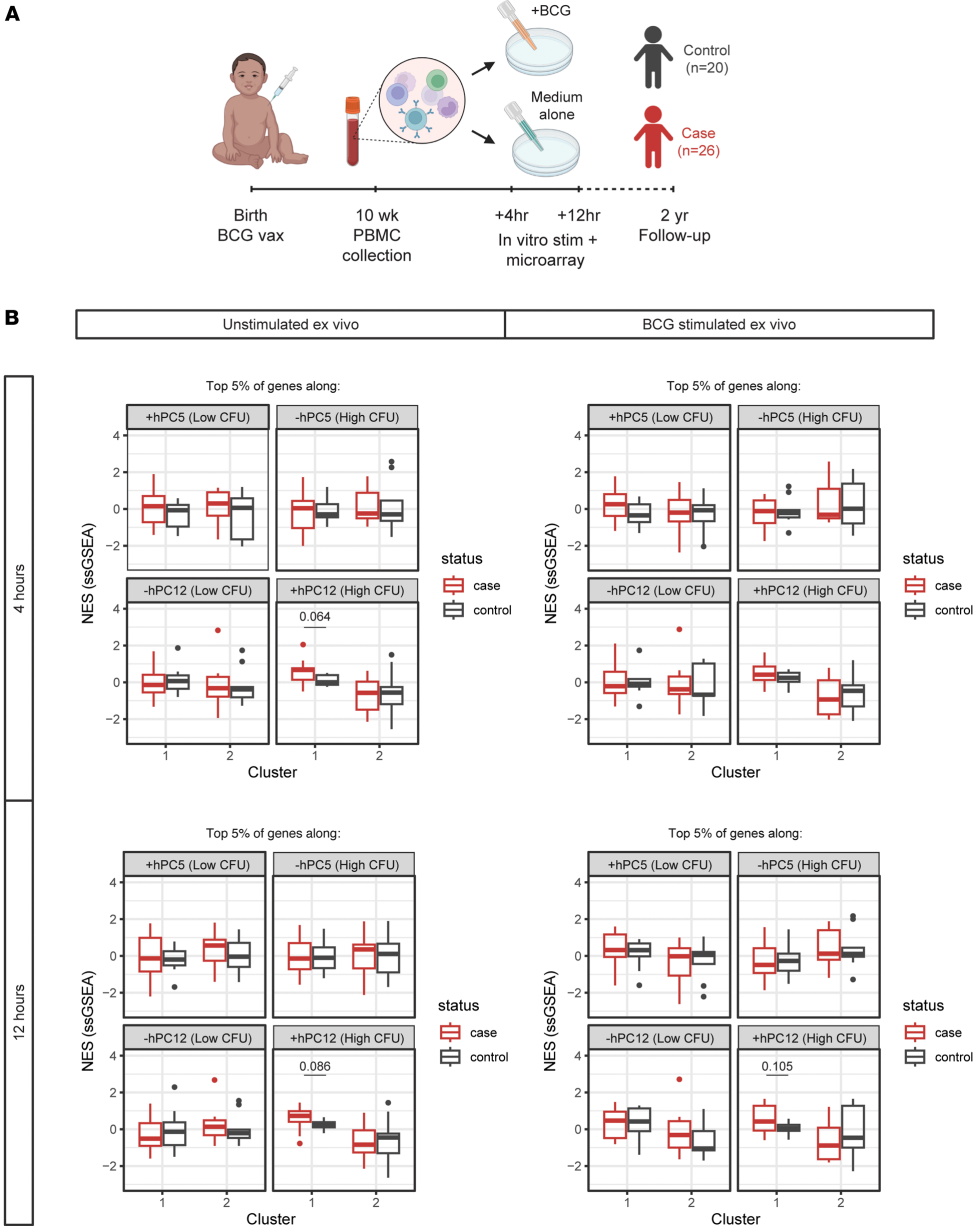
GSE20716 provides partial validation for associations between hPCs and protection. (**A**) Schematic detailing microarray data collection strategy for independent cohort of South African infants. (**B**) Box plots visualizing normalized enrichment scores (NESs) calculated with ssGSEA for each microarray sample either (left) left unstimulated or (right) BCG-stimulated ex vivo for (top) 4 or (bottom) 12 hours. The thick center line of the box denotes the median; the bounds of the boxes (hinges) denote the 25th and 75th percentiles; the upper and lower whiskers extend ±1.5 **×** interquartile range from the upper and lower hinges, respectively; data beyond the whiskers are considered outliers and plotted individually. Samples were separated by case (red) versus control (blue) follow-up outcome and by major cluster assigned in the original publication of this dataset. Statistical testing between distributions of enrichment scores between cases and controls was conducted by unpaired *t* test.

Finally, because our cross-species analyses allowed us to generate hypotheses about transcriptional correlates of protection in vaccinated infants, we wanted to approach their validation. Although vaccine-induced protection is difficult to ascertain in humans, Fletcher et al. (35) previously published a microarray dataset that captured PBMCs, either left unstimulated or stimulated ex vivo with BCG, from South African infants who were vaccinated with BCG at birth and who later either developed TB (cases) or remained disease-free (controls) during 2 years of follow-up (Figure 5A and Supplemental Table 4). While the original study failed to reliably identify a correlate of risk, the authors instead classified patients into 2 clusters by hierarchically clustering samples by their respective differences in BCG-stimulated and unstimulated gene expression at the 12-hour time point only. The authors observed that immune phenotypes presented differently in cases depending on their cluster membership. Therefore, we were interested to use this microarray dataset with case and control outcomes as a validation set. To do this, we calculated enrichment scores for each sample using the top 5% of genes along each extreme of hPC5 and hPC12 and compared these scores across clusters and follow-up outcomes for each ex vivo stimulation condition (Figure 5B and Supplemental Table 5). Although no meaningful separations were observed by hPC5 gene expression, we noted, for cluster 1 only, that cases and controls could be nearly distinguished by expression of genes in the top 5% of the positive end of the hPC12 axis, i.e., (+) hPC12 across most individual ex vivo stimulation conditions. Using Fisher’s method to merge *P* values across stimulation and time point pairs (4 conditions total) indeed yielded statistical significance for this comparison only (*P* = 0.027; see Supplemental Table 6). We repeated these analyses for hPCs found to be associated with protection in only one NHP cohort (Supplemental Figure 8 and Supplemental Table 6), which further confirmed that the greatest separation between cases and controls was achieved using hPC12 genes. These results lent support for the idea that our species translation modeling can leverage animal model data to identify correlates of relevant human phenotypes that can be difficult to assess using human data alone.

## Discussion

BCG is currently the only TB vaccine available. Despite having been first approved for clinical use over a century ago (2), our understanding of the mechanism(s) of action of BCG and the limitations of its efficacy remain elusive (3–5), likely due in part to constraints on disease characterization imposed by present clinical diagnostic tools (10, 11, 49). Animal models including mice and NHPs have thus been employed to investigate BCG-mediated protective immunity, but these models can further complicate the identification of human-translatable biological insights due to cross-species discrepancies in the phenotypic relevance of immune processes and pathways (17, 18, 21).

Our lab has developed a cross-species modeling framework called TransCompR (27) that evaluates orthogonal axes of transcriptional or proteomic variability in one species with respect to a phenotype or biological outcome of interest in another species, allowing for the explicit accounting of cross-species differences. Previous applications of TransCompR have identified underappreciated pathological mechanisms in various disease contexts that were not apparent with traditional cross-species comparisons (21, 27, 29–32). Here, we adapted TransCompR to identify human-relevant biological pathways that were predictive of BCG-mediated protective immunity, a disease-relevant biological outcome that is best characterized in animal models given experimental and ethical considerations. We used both newly generated and publicly available (33, 34) bulk blood transcriptomics datasets from human participants and animal models to construct and test our species translation model. It is important to note that lung-localized immune responses, collected at the site of infection and typically quantified via BAL, might be more disease-relevant. However, blood-based responses maintain clinical relevance because they are more easily measured at scale in human participants. We also note that the human data captured transcriptional responses to percutaneous and intradermal BCG vaccination, while much of the animal model data described responses to intravenous BCG vaccination, a route that is not approved for clinical use. Different routes of BCG vaccination, despite reported differences in protective efficacy, have been shown to induce many of the same cellular responses in BAL (34) and humoral responses in plasma (50), albeit at varying magnitudes, supporting the validity of our cross-species comparison of datasets.

Our hPC model, built on human blood transcriptomic data, was separately able to predict ex vivo BCG-specific immune responses in infants (Figure 2) and post–*M*. *tuberculosis* challenge outcomes, as assessed in two different publicly available, BCG-vaccinated NHP cohorts (Figure 3). Interestingly, we found no hPCs that were associated both with protection across the 2 NHP cohorts and BCG-specific T cell responses in infants (Supplemental Figure 5D). This result is somewhat surprising given that antigen-specific IFN-γ production by T cells has been historically considered to be a BCG-mediated immune correlate of protection (22, 51), although there is a growing body of literature that contradicts this in both humans and animal models (6, 52). The magnitude of *M*. *tuberculosis*–specific IFN-γ production has also been previously proposed as a marker of disease progression (53), which might explain why the nonprotected end of hPC4 corresponded with BCG-specific IFN-γ expression in T cells (Figure 2C and Figure 3, B and C), and provides a plausible mechanism for BCG-specific Th1 subsets that fail to protect. It is also possible that BCG-mediated antimycobacterial immunity requires polyfunctional T cell responses, which have been demonstrated by previous literature (54) and which are not captured by observing IFN-γ expression alone.

Instead, we observed a statistically significant association between hPC12 and BCG-reactive IL-6 expression in mDCs. IL-6 is a pleiotropic cytokine with a wide array of biological functions. In the contexts of BCG vaccination and *M*. *tuberculosis* infection, most previous literature has focused on establishing the role of IL-6 in CD4^+^ (Th1/17) and CD8^+^ T cell activation (55, 56). However, the observed antigen “specificity” of this mDC response, given that hPC12 maintained no significant associations with IL-6^+^ mDCs (%) under unstimulated or SEB-stimulated conditions (Supplemental Figure 1B), was unexpected given that it is an innate immune response. This finding bears similarity to findings from recent work demonstrating BCG-induced innate immune memory, also called trained immunity, which occurs via epigenetic reprogramming and has been observed in various cell types, including bone marrow hematopoietic stem cells, multipotent progenitors, and circulating monocytes (57–60). This phenomenon has also been observed in mature myeloid cell types, including lung-resident alveolar macrophages with BCG vaccination (61) and splenic DCs with fungal pathogenic stimulation (62). More evidence is needed to definitively disentangle whether DCs can be induced to elicit memory-like immunological responses against mycobacteria and how these responses might participate in BCG-mediated protective immunity.

Of those human-derived PCs found to associate with protection in NHPs, a common theme of time dependence emerged. This was a fundamental nuance of our analyses, which emphasized the dynamic nature of vaccine-induced processes and pathways and highlighted the importance of considering time as a variable when assessing BCG-mediated immune correlates of protection. On this topic, we further noted that the temporal component of the associations between hPC5 and hPC12 and protection was the same for both cohorts (Figure 3B and Supplemental Figure 5B), lending further support for the idea that these orthogonal axes of transcriptional variance represented species-translatable, time-dependent, and largely acute mechanisms that play a role in BCG-mediated protective immunity.

We next used GSEA to reveal which biological processes and pathways might be captured by the protection-associated human-derived PCs in our species translation model, focusing our interpretation on hPC12 and hPC5. hPC12, across reference databases (39, 40), was enriched for innate immune activation via RIG-I–like and NOD-like receptors, antiviral immunity, and type I IFN signaling. RIG-I agonism in concert with BCG vaccination has been shown, albeit in mice, to enhance antigen presentation, myeloid-specific β secretion, memory T cell expansion, and BCG-mediated protective responses (63), and therefore, it is possible that hPC12 captures transcriptional variability associated with multiple innate immune processes that ultimately cooperate to facilitate anti-TB immunity through the initiation of mycobacterial-specific T cell responses. The characterization of type I IFN signaling as protective might be controversial given that previous studies have described this pathway as an immune correlate of risk (64, 65); however, timing and vaccination status might provide crucial context, as other studies have described a protective role for type I IFNs against *M*. *tuberculosis* infection specifically during the acute phase of response to BCG vaccination (33, 66). This dual role of type I IFNs has also been observed in response to viral vaccines (67, 68).

Regarding hPC5, we found this human-derived axis of transcriptional variability to be enriched for biological processes concerning innate-adaptive immune crosstalk, Th differentiation, and B cell activation. Of particular interest was the enrichment of hPC5 for signaling via Dectin-1, which is typically restricted to APCs, and has been previously linked to antimycobacterial immune responses in part through the induction of Th1/Th17-polarizing cytokines (43–46). While antigen-specific Th1 responses have long been considered to be necessary for natural and BCG-induced anti-TB immunity (69–71), recent work has described roles for noncanonical T cell subsets, including Th17 cells, in *M*. *tuberculosis* control (32, 72) and in vaccine-induced protective responses (73–75) across both humans and animal models. hPC5 being enriched for Th17 differentiation and Th17-inducible cytokine signaling thus provides support for the idea that noncanonical T cell responses should be further considered as a species-translatable feature of BCG-mediated protective immunity. Moreover, evaluating the enrichment of these gene sets over time with ssGSEA (Figure 4D and Supplemental Figure 7) reinforced our assertions that the human-relevant correlates of protection identified by our species translation model were BCG-induced and translatable even across different animal model species, albeit not time-agnostic.

Finally, we partially validated the protection-associated mechanisms inferred from our translation model using a publicly available microarray dataset collected from BCG-vaccinated South African infants, which were categorized as cases or controls during 2 years of follow-up (35). The original study, which was unable to robustly characterize a common correlate of risk from the microarray data, instead identified 2 clusters of infants across follow-up outcomes by their patterns in gene expression. Of these 2 clusters, we found that infants belonging to cluster 1 could be distinguished as cases or controls by their blood transcriptional expression of genes in the top 5% of the positive end of the hPC12 axis, i.e., (+) hPC12 by loadings. Expression of genes along either extreme of hPC5, which we also predicted to be associated with protection, did not significantly separate infants in this cohort with respect to their follow-up outcome. Partial validation of our hypotheses was not a surprising result, given that existing clinical data that attempt to characterize BCG-mediated protection outcomes are quite limited. Vaccination at birth is standard of care in countries with high TB incidence and precludes collection of data from nonvaccinated participants, which limits the ability to distinguish between vaccine-induced and natural anti-TB immunity. Additionally, without deliberate pathogen exposure, it is possible that healthy controls comprise both protected and unexposed individuals, which would further occlude the detection of BCG-mediated protective signals. Moreover, as demonstrated in Fletcher et al. (35), variance among study participants can yield divergent host responses to vaccination and mask disease-relevant biological signals. We further note that validation was more frequently achieved in unstimulated rather than BCG-stimulated samples, although this could be attributed in part to technical artifacts introduced by the substantial experimental manipulation required to thaw and restimulate cryopreserved samples with a live bacterium. Regardless of limitations in the validation data, we also acknowledge the caveat that our species translation modeling approach identifies human-relevant, i.e., conserved, signatures of protection from NHP data, and human relevance is not alone a sufficient condition for disease relevance in humans.

Overall, this study highlights the utility of translational cross-species modeling to leverage animal studies to uncover mechanisms of action in humans where no outcome data are available. While applied here to analyze human and NHP bulk blood transcriptomics to investigate BCG-mediated protective immunity, TransCompR is generalizable to a wide variety of biological contexts, species, and data modalities, although its reliance on homology matching does impose limitations on its application to cases where a 1-1 mapping between cross-species features does not exist. Despite this, our computational framework remains well-suited for heterogeneous and highly variable datasets, facilitating a systems biology approach to identifying disease-relevant biological variance. Altogether, cross-species modeling is a useful tool to better understand the mechanisms underlying vaccine-mediated protective immunity, ultimately supporting the development of improved preventive interventions for TB.

## Methods

### Sex as a biological variable

Of the South African infants profiled by bulk RNA-seq (*n* = 60), 29 were male and 31 were female. We observed no significant associations between human-derived PCs retained in the species translation model and biological sex (results not shown), and therefore we assert similar findings for both sexes. The publicly available NHP cohorts were well-balanced regarding biological sex, and sex-dimorphic effects were not observed with respect to post–*M*. *tuberculosis* challenge outcomes. Regarding the DO mice, our study exclusively examined female DO mice owing to issues with aggression and housing constraints experienced with male mice.

### Cohorts and corresponding study designs

#### South African infant cohort.

We performed a nested case-control study of participants from a parent cohort of 11,680 South African infants (6, 7), who were vaccinated with BCG either percutaneously or intradermally within 24 hours of birth and from whom blood was collected at 10 weeks after birth for immunological analyses (6). The vast majority (88.33%) of these infants were of mixed ethnic ancestry, with a median birth weight of 2.81 kg. In this work, we analyzed a group of 60 infants (29 male, 31 female) who had available cryopreserved PBMCs and who had the highest or lowest IFN-γ–expressing T cell responses to BCG, selected from 189 infants with data on BCG-specific cytokine responses (76). Selection of these infants was not informed by biological sex.

#### NHP dose cohort (GSE218270).

34 adult Indian-origin rhesus macaques (*M*. *mulatta*) (16 male, 18 female; median age 4.4 years) were included in the previously published dose cohort (33). Macaques were randomized into 6 vaccine groups to receive varying doses of intravenous BCG vaccination (4.5–7.5 log_10_ CFU, in half-log increments), and whole blood was collected 4 weeks prior to vaccination and at 2 days, 2 weeks, 4 weeks, and 12 weeks after vaccination for bulk RNA-seq. Macaques were challenged 5–6 months after vaccination with a low dose of *M*. *tuberculosis* and euthanized 12 weeks later, or at the humane endpoint, for analysis of disease burden. As in the original publication (33), protection was defined as fewer than 100 total CFU *M*. *tuberculosis* in lung tissues upon necropsy. Processed sequencing data were obtained from GEO under accession number GSE218270. Corresponding flow cytometry and antibody titers measured at the same time points were obtained from a repository (https://github.com/Khatri-Lab/bcg_transcriptome; branch name: main; Commit ID: c14215d…8df49c6).

#### NHP route cohort (GSE218157).

35 adult Indian-origin rhesus macaques (*M*. *mulatta*) (17 male, 18 female; median age 4.7 years) were included in the previously published route cohort (36). Macaques received BCG vaccination through different routes that were randomly assigned: aerosol (*n* = 7), intradermal (*n* = 7), high-dose intradermal (*n* = 8), combined aerosol and intradermal (*n* = 7), and intravenous at 5 × 10^7^ CFU of BCG (*n* = 7). Whole blood was collected at baseline and at 2 days, 2 weeks, and 12 weeks after vaccination for bulk RNA-seq. Macaques were later challenged with *M*. *tuberculosis* 6–10 months following BCG vaccination and euthanized 11–15 weeks following challenge or at humane endpoint for analysis of disease burden. As in the original publication, protection was defined as fewer than 100 total CFU *M*. *tuberculosis* [i.e., log_10_(CFU) < 2] in lung tissues upon necropsy. Processed sequencing data were obtained from GEO under accession number GSE218157.

### DO mouse cohort

DO mice were purchased from The Jackson Laboratory. Whole blood was collected from 99 DO mice (48) 2 weeks prior to and 2–3 weeks postintradermal BCG (Pasteur) vaccination for bulk RNA-seq. Mice were then challenged with aerosol *M*. *tuberculosis* infection 8 weeks after vaccination and euthanized 14 weeks following challenge for analysis of disease burden. Protection was defined as fewer than 25,000 total CFU *M*. *tuberculosis* [i.e., log_10_(CFU) < 4.4] in lung tissues upon necropsy. Nine mice died prior to endpoint, and their disease burdens were unable to be assessed; these mice were excluded from our analyses.

#### Fletcher et al. cohort (GSE20716).

46 BCG-vaccinated South African infants were included in the previously published microarray dataset from Fletcher et al. (35). Blood was collected 10 weeks after birth for PBMC isolation and microarray data analysis. Infants were then followed over a 2-year period for characterization of confirmed TB cases (*n* = 26) or controls (*n* = 20). Processed microarray data were obtained from GEO under accession number GSE20716.

### Human blood collection, stimulation, and cryopreservation

At 10 weeks of age, heparinized venous blood was collected from all infants. For ICS, 1 mL whole blood was immediately incubated with BCG (SSI strain, 1.2 × 106 organisms/mL), as previously described (6). Whole blood incubated with medium alone served as negative control, and SEB (10 μg/mL; Sigma-Aldrich) was used as positive control. The costimulatory antibodies, anti-CD28 (L293) and anti-CD49d (L25) (1 μg/mL each; BD Biosciences), were added to all conditions for enhancement of specific responses. Blood was incubated for 7 hours at 37°C. Brefeldin-A was then added, followed by incubation for an additional 5 hours. Cells were then harvested, fixed, and cryopreserved as previously described (77). PBMCs were isolated by Ficoll-density gradient centrifugation and cryopreserved in liquid nitrogen. For bulk RNA-seq, PBMCs were thawed, washed, and immediately used for RNA extraction.

### Human bulk RNA-seq and analysis

RNA was isolated according to the manufacturer’s protocol using an miRNeasy Mini Kit (Qiagen). RNA integrity and yield was tested on the Agilent 2100 Bioanalyzer. The resulting cDNA was used to prepare a sequencing library. Libraries were sequenced on a MGI DNBSEQ-G400RS platform using DNBSEQ-G400RS high through-put sequencing set (FCL PE100) to acquire paired-end reads of 100 base pairs in length. Reads were aligned against the human reference genome: Homo sapiens GRCh38. Analysis of reads was performed in R v4.1.2. The data were log_2_-normalized (voom transformation) for downstream analyses. Lowly expressed genes were filtered by CPM greater than 2 in at least 5 samples.

### Human ICS

ICS was performed as previously described (76). Briefly, cryopreserved cells were thawed, washed, and permeabilized with Perm/wash solution (BD Biosciences). Cells were then incubated at 4°C for 1 hour with fluorescence-conjugated antibodies directed against surface antigens and intracellular cytokines. The following antibodies from BD Biosciences were used: anti-CD3 APC-H7 (clone SK7); anti-CD66 BV711 (B6.2/CD66); anti-CD14 PercP eFluor 710 (61D3); anti-CD33 BV650 (WM5); anti-CD16 AF488 (3G); anti-CD123 BV785 (6H6); anti-CD11c BV421 (3.9); anti–TNF-α Cy7PE (Mab11); anti–IFN-γ AF700 (B27); anti-IL-6 APC (MQ2-13A5); anti-PD-L1/CD274 PE (MIH1); and anti-CD40 BV510 (5C3). Cells were acquired on a LSR II flow cytometer (BD Biosciences) configured with 3 lasers and 10 detectors, with FACS Diva 6.1 software. Optimal photomultiplier tube settings were established for this study before sample analysis. Cytometer setting and tracking beads (BD Biosciences) were used to record the target median fluorescence intensity values for the baseline settings, and these calibrations were performed each day before sample acquisition. Compensation settings were set with anti-mouse κ-beads (BD Biosciences) labeled with the respective fluorochrome-conjugated antibodies. Flowjo version 8.8.4 (Treestar) was used to compensate and to analyze the flow cytometric data.

### Mouse vaccinations, bacterial infections, and assessment of bacterial burden

100 DO mice were vaccinated intradermally with 10^5^ CFU of BCG. At 8 weeks after vaccination, animals were challenged by aerosol with *M*. *tuberculosis* Erdman with –50 CFU delivered to the lungs. Bacterial burdens in lung tissue were determined at necropsy at 14 weeks after challenge as previously described (78). These 100 animals were chosen from replicate experiments of similar design and outcome from previously published studies (79) and represented a spectrum of outcomes regarding lung and spleen *M*. *tuberculosis* burden, lung histopathology, and early moribund.

### Mouse blood collection for bulk RNA-seq

For each DO mouse, approximately 100 μL blood was collected via tail nick directly into RNALater (Thermo Fisher Scientific) at 14 days before and 14 days after vaccination. RNA was extracted from whole blood using the Mouse Ribopure Blood RNA kit (Ambion). All RNA samples were treated with DNase to remove contaminating DNA and then purified using QiaQuick Spin skits (Qiagen). RNA quantification was performed with a Nanodrop spectrophotometer (Thermo Fisher Scientific).

### Mouse bulk RNA-seq and analysis

RNA-seq was performed by Qiagen using 10 ng RNA per sample. Library preparation was done using the QIAseq UPX 3’ Transcriptome kit (Quiagen). During reverse transcription, each cell was tagged with a unique ID (up to 384 different IDs), and each RNA molecule is tagged with a unique molecular index (UMI). Then RNA was converted to cDNA. The cDNA was amplified using PCR and indices were added. After PCR, the samples were purified, and library preparation QC was performed using TapeStation 4200 (Agilent) or Agilent Bioanalyzer. Based on the quality of the inserts and the concentration measurements, the libraries were pooled in equimolar ratios. The library pool(s) were quantified using qPCR. The library pools were then sequenced on a NextSeq500 sequencing instrument according to the manufacturer instructions. Raw data were then processed using UMI-tools (80) to extract sample barcodes, remove globin transcripts in silico (>90% total RNA), and perform gene-wise deduplication. FASTQ files for each sample were then generated using bam2fastq. Genome Reconstruction by RNA-seq (81) was then used to perform haplotype-specific alignment of reads against a transcriptome reference containing sequences from all 8 parental strains to generate a count matrix of reads assigned to their most likely origins. Samples with fewer than 2,000 total counts were removed for downstream analysis, and then a ZINB-WaVE transformation (82) was applied to the data in place of normalization.

### Regression of principal component scores onto covariates

Univariate regressions were performed to identify covariates that explain variation in score distributions on specific hPCs. For binary covariates (human: responder group; NHP: binned postchallenge protection outcome), logistic regressions were performed, and *z* values were reported as regression coefficients. For continuous covariates (human: ICS features; NHP: BCG dose, postchallenge lung bacterial burden), linear regressions were performed, and *t* values were reported as regression coefficients. All regression coefficients are presented as average ± SD from 5-fold cross-validation.

### TransCompR

PCA was performed on the *z*-score-normalized, ortholog-limited human data using the prcomp function from the stats package (v3.6.2) in R to obtain the human sample scores and gene loadings on each hPC. The first 15 hPCs explained 80% of variance in the human data and were thus retained for downstream analyses. NHP samples were projected into this 15 hPC space by multiplying the *z*-score-normalized, ortholog-limited NHP data matrix by the human gene loadings matrix (Supplemental Table 7), resulting in a matrix of NHP scores in the hPC space. The variance of the NHP scores along all hPCs was totaled and used to normalize the amount of variance in the projected NHP data explained by each individual hPC. hPCs were then downselected by whether their univariate regression coefficient was statistically significant across all 5 cross-validation folds. Univariate regression modeling was conducted using the glm function from the stats package (v3.6.2) in R.

### TransCompR model validation

To ensure that we constructed a TransCompR model which predicted post–*M*. *tuberculosis* challenge bacterial burden with better performance than random chance, we compared prediction error or accuracy for covariate TransCompR models using all significantly univariately-associated hPCs to a series of null models with either random hPC selection or shuffled outcome variable (CFU or binned protection outcome). For all models, distributions of test error or accuracy scores were generated using 1,000 trials of 5-fold cross-validation. Distributions were then statistically compared by Wilcoxon’s rank-sum testing.

### GSEA

To biologically interpret protection-associated hPCs, we used the Reactome Pathway (39) and KEGG (40) databases as references. For Reactome Pathway terms, we used the fgsea package (v1.21.1) in R (83) to calculate enrichment scores and estimate *P* values. KEGG terms were limited to exclude those that were related to cancer or were specific to nonblood tissues. To calculate odds ratios for KEGG terms, we used the enrichR package (v3.3) in R (84), using the top 20% of genes along the extreme of each hPC as input. ssGSEA was performed using select Reactome Pathway leading edge gene sets, and statistical testing between relevant groups (protected vs. nonprotected) was conducted by unpaired *t* test with Holm-Šídák’s multiple comparison correction across time points calculated in GraphPad.

### Statistics

Summary data are presented as mean ± SD unless otherwise specified. Statistical analyses are specified in the figure legends and include unpaired 2-tailed *t* test, Holm-Šídák’s multiple comparison correction, univariate regression, and Wald’s test. Values were generally considered significant at *P* < 0.05. All analyses were performed using either custom R scripts or GraphPad Prism (version 10.0.3).

### Study approval

Human participants were enrolled from a parent study (6, 7), which was conducted in accordance with the US Department of Health and Human Services and Good Clinical Practice guidelines and included protocol approval by the University of Cape Town Human Research Ethics Committee (332/2005) and written informed consent from the parent or legal guardian. All studies utilizing DO mice were performed under protocols approved by the Institutional Animal Care and Use Committee of the Center for Biologics Evaluation and Research at the Food and Drug Administration (#2011-14). Animal protocols stressed practices and procedures designed to strictly minimize any suffering.

### Data availability

The publicly available data utilized in this study can be found on NCBI Gene Expression Omnibus (GEO) under the following accession numbers: GSE218270 (NHP dose cohort), GSE218157 (NHP route cohort), GSE20716 (infant microarray data). The BCG-vaccinated infant bulk RNA-seq data published in this study are publicly available in the Sequence Read Archive (SRA) with BioProject ID PRJNA1321723. The DO mouse bulk RNA-seq data published in this study are publicly available in GEO at GSE308309. The TransCompR species translation modeling code was adapted from MathWorks File Exchange (no. 77987). Values for all data points in graphs are reported in the Supporting Data Values file.

## Author contributions

KB, CMS, TJS, and DAL conceived the study. DA, AG, TDM, and SLK performed experiments. KB, DA, AG, TDM, SLK, and REB acquired and/or analyzed the data. KB wrote the original manuscript. KLE, CMS, TJS, and DAL supervised the study. CMS, TJS, and DAL acquired funding. All authors reviewed, edited, and agreed to the final version of the manuscript.

## Funding support

This work is the result of NIH funding, in whole or in part, and is subject to the NIH Public Access Policy. Through acceptance of this federal funding, the NIH has been given a right to make the work publicly available in PubMed Central.

- NIH contract 75N93019C00071 to CMS and DAL.
- NIH grant AI181898 to CMS, TJS, and DAL.
- Army Institute for Collaborative Biotechnologies collaborative agreement W911NF-19-2-0026 to DAL.
- National Institute of Environmental Health Sciences Training Grant in Environmental Toxicology T32-ES007020 to KB.

## Supplementary Material

Supplemental data

Supplemental table 3

Supplemental table 7

Supporting data values

## Version 1 11/11/2025

In-Press Preview

## Version 2 12/22/2025

Electronic publication

## References

[1] https://www.who.int/teams/global-programme-on-tuberculosis-and-lung-health/tb-reports/global-tuberculosis-report-2024.

[2] Davenne T, McShane H (2016). Why don’t we have an effective tuberculosis vaccine yet?. Expert Rev Vaccines.

[3] Martinez L (2022). Infant BCG vaccination and risk of pulmonary and extrapulmonary tuberculosis throughout the life course: a systematic review and individual participant data meta-analysis. Lancet Glob Health.

[4] Roy A (2014). Effect of BCG vaccination against Mycobacterium tuberculosis infection in children: systematic review and meta-analysis. BMJ.

[5] Mangtani P (2014). Protection by BCG vaccine against tuberculosis: a systematic review of randomized controlled trials. Clin Infect Dis.

[6] Kagina BMN (2010). Specific T cell frequency and cytokine expression profile do not correlate with protection against tuberculosis after bacillus Calmette-Guérin vaccination of newborns. Am J Respir Crit Care Med.

[7] Hawkridge A (2008). Efficacy of percutaneous versus intradermal BCG in the prevention of tuberculosis in South African infants: randomised trial. BMJ.

[8] Zak DE (2016). A blood RNA signature for tuberculosis disease risk: a prospective cohort study. Lancet.

[9] Huang Y (2022). Review and updates on the diagnosis of tuberculosis. J Clin Med.

[10] MacGregor-Fairlie M (2020). Tuberculosis diagnostics: overcoming ancient challenges with modern solutions. Emerg Top Life Sci.

[11] Chopra KK, Singh S (2020). Newer diagnostic tests for tuberculosis, their utility, and their limitations. Curr Med Res Pract.

[12] Wallis RS (2009). Biomarkers for tuberculosis disease activity, cure, and relapse. Lancet Infect Dis.

[13] Doherty M (2009). Biomarkers for tuberculosis disease status and diagnosis. Curr Opin Pulm Med.

[14] Russell DG (2010). Tuberculosis: what we don’t know can, and does, hurt us. Science.

[16] Singh AK, Gupta UD (2018). Animal models of tuberculosis: lesson learnt. Indian J Med Res.

[17] Seyhan AA (2019). Lost in translation: the valley of death across preclinical and clinical divide – identification of problems and overcoming obstacles. Transl Med Commun.

[18] Hackam DG, Redelmeier DA (2006). Translation of research evidence from animals to humans. JAMA.

[19] Shay T (2013). Conservation and divergence in the transcriptional programs of the human and mouse immune systems. Proc Natl Acad Sci U S A.

[20] Godec J (2016). Compendium of immune signatures identifies conserved and species-specific biology in response to inflammation. Immunity.

[21] Pullen KM (2025). Cross-species transcriptomics translation reveals a role for the unfolded protein response in Mycobacterium tuberculosis infection. NPJ Syst Biol Appl.

[22] Abebe F (2012). Is interferon-gamma the right marker for bacille Calmette-Guérin-induced immune protection? The missing link in our understanding of tuberculosis immunology. Clin Exp Immunol.

[23] Andersen P, Smedegaard B (2000). CD4(+) T-cell subsets that mediate immunological memory to Mycobacterium tuberculosis infection in mice. Infect Immun.

[24] Caruso AM (1999). Mice deficient in CD4 T cells have only transiently diminished levels of IFN-gamma, yet succumb to tuberculosis. J Immunol.

[25] Soares AP (2008). Bacillus Calmette-Guérin vaccination of human newborns induces T cells with complex cytokine and phenotypic profiles. J Immunol.

[26] Normand R (2018). Found in translation: a machine learning model for mouse-to-human inference. Nat Methods.

[27] Brubaker DK (2020). An interspecies translation model implicates integrin signaling in infliximab-resistant inflammatory bowel disease. Sci Signal.

[28] Kowald A (2022). Transfer learning of clinical outcomes from preclinical molecular data, principles and perspectives. Brief Bioinform.

[29] Suarez-Lopez L (2021). Cross-species transcriptomic signatures predict response to MK2 inhibition in mouse models of chronic inflammation. iScience.

[30] Lee MJ (2021). Computational interspecies translation between Alzheimer’s disease mouse models and human subjects identifies innate immune complement, TYROBP, and TAM receptor agonist signatures, distinct from influences of aging. Front Neurosci.

[31] Carroll MJ (2021). Translatable pathways classification (TransPath-C) for inferring processes germane to human biology from animal studies data: example application in neurobiology. Integr Biol (Camb).

[32] Proulx MK (2025). Noncanonical T cell responses are associated with protection from tuberculosis in mice and humans. J Exp Med.

[33] Liu YE (2023). Blood transcriptional correlates of BCG-induced protection against tuberculosis in rhesus macaques. Cell Rep Med.

[34] Darrah PA (2020). Prevention of tuberculosis in macaques after intravenous BCG immunization. Nature.

[35] Fletcher HA (2016). Human newborn bacille Calmette-Guérin vaccination and risk of tuberculosis disease: a case-control study. BMC Med.

[36] Darrah PA (2023). Airway T cells are a correlate of i.v. Bacille Calmette-Guerin-mediated protection against tuberculosis in rhesus macaques. Cell Host Microbe.

[37] Irvine EB (2024). Humoral correlates of protection against Mycobacterium tuberculosis following intravenous BCG vaccination in rhesus macaques. iScience.

[38] Meimetis N (2024). AutoTransOP: translating omics signatures without orthologue requirements using deep learning. NPJ Syst Biol Appl.

[39] Milacic M (2024). The reactome pathway knowledgebase 2024. Nucleic Acids Res.

[40] Kanehisa M (2025). KEGG: biological systems database as a model of the real world. Nucleic Acids Res.

[41] Ahmed M (2020). Immune correlates of tuberculosis disease and risk translate across species. Sci Transl Med.

[42] Rosa BA (2021). IFN signaling and neutrophil degranulation transcriptional signatures are induced during SARS-CoV-2 infection. Commun Biol.

[43] Mata-Martínez P (2022). Dectin-1 signaling update: new perspectives for trained immunity. Front Immunol.

[44] Rothfuchs AG (2007). Dectin-1 interaction with Mycobacterium tuberculosis leads to enhanced IL-12p40 production by splenic dendritic cells. J Immunol.

[45] Yadav M, Schorey JS (2006). The beta-glucan receptor dectin-1 functions together with TLR2 to mediate macrophage activation by mycobacteria. Blood.

[46] Wagener M (2018). Dectin-1-Syk-CARD9 signaling pathway in TB immunity. Front Immunol.

[47] Zhu X, Zhu J (2020). CD4 T helper cell subsets and related human immunological disorders. Int J Mol Sci.

[48] Bogue MA (2015). Collaborative cross and diversity outbred data resources in the mouse phenome database. Mamm Genome.

[49] Pai M (2016). Tuberculosis diagnostics: state of the art and future directions. Microbiol Spectr.

[50] Irvine EB (2021). Robust IgM responses following intravenous vaccination with Bacille Calmette-Guérin associate with prevention of Mycobacterium tuberculosis infection in macaques. Nat Immunol.

[51] Fletcher HA (2016). T-cell activation is an immune correlate of risk in BCG vaccinated infants. Nat Commun.

[52] Mittrücker H-W (2007). Poor correlation between BCG vaccination-induced T cell responses and protection against tuberculosis. Proc Natl Acad Sci U S A.

[53] Andersen P (2007). The prognosis of latent tuberculosis: can disease be predicted?. Trends Mol Med.

[54] Smith SG (2016). Polyfunctional CD4 T-cells correlate with in vitro mycobacterial growth inhibition following Mycobacterium bovis BCG-vaccination of infants. Vaccine.

[55] Bizzell E (2018). Deletion of BCG Hip1 protease enhances dendritic cell and CD4 T cell responses. J Leukoc Biol.

[56] Su H (2019). The Mycobacterium tuberculosis glycoprotein Rv1016c protein inhibits dendritic cell maturation, and impairs Th1 /Th17 responses during mycobacteria infection. Mol Immunol.

[57] Kleinnijenhuis J (2012). Bacille Calmette-Guerin induces NOD2-dependent nonspecific protection from reinfection via epigenetic reprogramming of monocytes. Proc Natl Acad Sci U S A.

[58] Moorlag SJCFM (2024). Multi-omics analysis of innate and adaptive responses to BCG vaccination reveals epigenetic cell states that predict trained immunity. Immunity.

[59] Li W (2023). A single-cell view on host immune transcriptional response to in vivo BCG-induced trained immunity. Cell Rep.

[60] Sun SJ (2024). BCG vaccination alters the epigenetic landscape of progenitor cells in human bone marrow to influence innate immune responses. Immunity.

[61] Jeyanathan M (2022). Parenteral BCG vaccine induces lung-resident memory macrophages and trained immunity via the gut-lung axis. Nat Immunol.

[62] Hole CR (2019). Induction of memory-like dendritic cell responses in vivo. Nat Commun.

[63] Khan A (2020). NOD2/RIG-I activating inarigivir adjuvant enhances the efficacy of BCG vaccine against tuberculosis in mice. Front Immunol.

[64] Satti I (2022). Inflammation and immune activation are associated with risk of Mycobacterium tuberculosis infection in BCG-vaccinated infants. Nat Commun.

[65] Donovan ML (2017). Type I interferons in the pathogenesis of tuberculosis: molecular drivers and immunological consequences. Front Immunol.

[66] Moreira-Teixeira L (2018). Type I interferons in tuberculosis: foe and occasionally friend. J Exp Med.

[67] Zhong C (2021). Type I interferon promotes humoral immunity in viral vector vaccination. J Virol.

[68] Palacio N (2020). Early type I IFN blockade improves the efficacy of viral vaccines. J Exp Med.

[69] Bustamante J (2014). Mendelian susceptibility to mycobacterial disease: genetic, immunological, and clinical features of inborn errors of IFN-γ immunity. Semin Immunol.

[70] Boisson-Dupuis S (2018). Tuberculosis and impaired IL-23-dependent IFN-γ immunity in humans homozygous for a common TYK2 missense variant. Sci Immunol.

[71] Marchant A (1999). Newborns develop a Th1-type immune response to Mycobacterium bovis bacillus Calmette-Guérin vaccination. J Immunol.

[72] Sun M (2024). Specific CD4^+^ T cell phenotypes associate with bacterial control in people who ‘resist’ infection with Mycobacterium tuberculosis. Nat Immunol.

[73] Singhania A (2021). CD4+CCR6+ T cells dominate the BCG-induced transcriptional signature. eBioMedicine.

[74] Wozniak TM (2010). Mycobacterium bovis BCG-specific Th17 cells confer partial protection against Mycobacterium tuberculosis infection in the absence of gamma interferon. Infect Immun.

[75] Gopal R (2012). IL-23-dependent IL-17 drives Th1-cell responses following Mycobacterium bovis BCG vaccination. Eur J Immunol.

[76] Anterasian C (2025). BCG induced innate immune response heterogeneity and susceptibility to pediatric tuberculosis. J Immunol.

[77] Hanekom WA (2004). Novel application of a whole blood intracellular cytokine detection assay to quantitate specific T-cell frequency in field studies. J Immunol Methods.

[78] Kurtz SL (2020). The diversity outbred mouse population is an improved animal model of vaccination against tuberculosis that reflects heterogeneity of protection. mSphere.

[79] Kurtz SL (2024). Multiple genetic loci influence vaccine-induced protection against Mycobacterium tuberculosis in genetically diverse mice. PLoS Pathog.

[80] Smith T (2017). UMI-tools: modeling sequencing errors in unique molecular identifiers to improve quantification accuracy. Genome Res.

[82] Risso D (2018). A general and flexible method for signal extraction from single-cell RNA-seq data. Nat Commun.

[84] Kuleshov MV (2016). Enrichr: a comprehensive gene set enrichment analysis web server 2016 update. Nucleic Acids Res.

