# Supplementary material for: Cross-species blood transcriptional correlates of BCG-mediated protection against tuberculosis include innate and adaptive immune processes
Source: JCI Insight. 2025 Nov 11;10(24):e194450. doi: 10.1172/jci.insight.194450 (PMC12890509; doi:10.1172/jci.insight.194450)
Supplement: Supplemental data [file jciinsight-10-194450-s150.pdf]

Supplemental Figure 1: ICS provides additional context for human-derived PCs

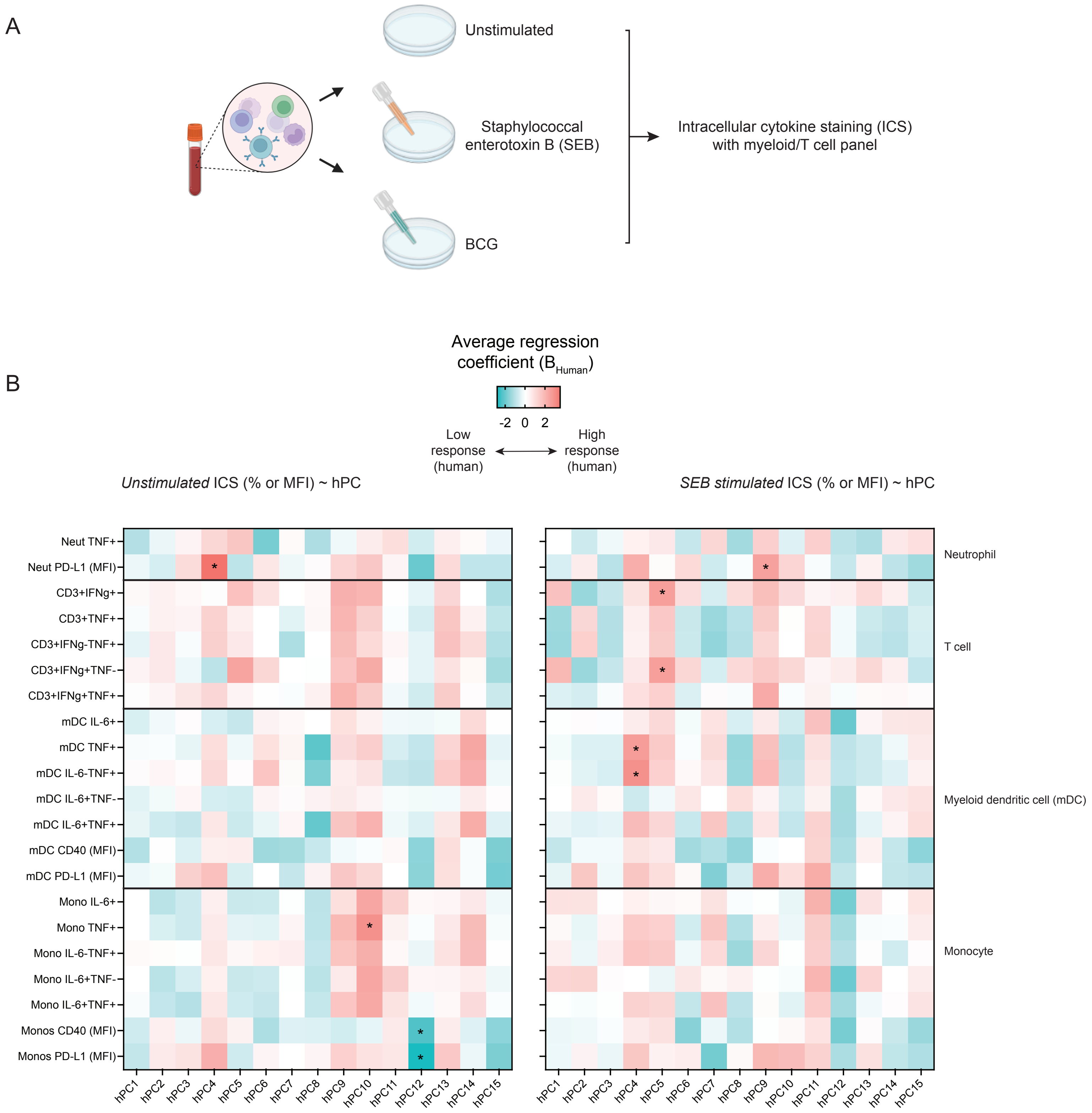

Related to Figs. 1 and 2.
(**A**) Schematic detailing collection of intracellular cytokine staining (ICS) data from infants profiled for bulk RNA-seq. SEB stimulation was used as a positive control to test for non-BCG-specific immune responses.
(**B**) Heatmaps detailing univariate regression coefficients from individual human-derived principal components regressed against ICS panel readouts (% unless otherwise indicated; y-axis) from unstimulated (left) and SEB-stimulated (right) *ex vivo* conditions. Regression coefficients are averaged across results from 5-fold cross validation. *p < 0.05 across folds.

Supplemental Figure 2: hPCs can separate human samples by BCG-specific Th1 response

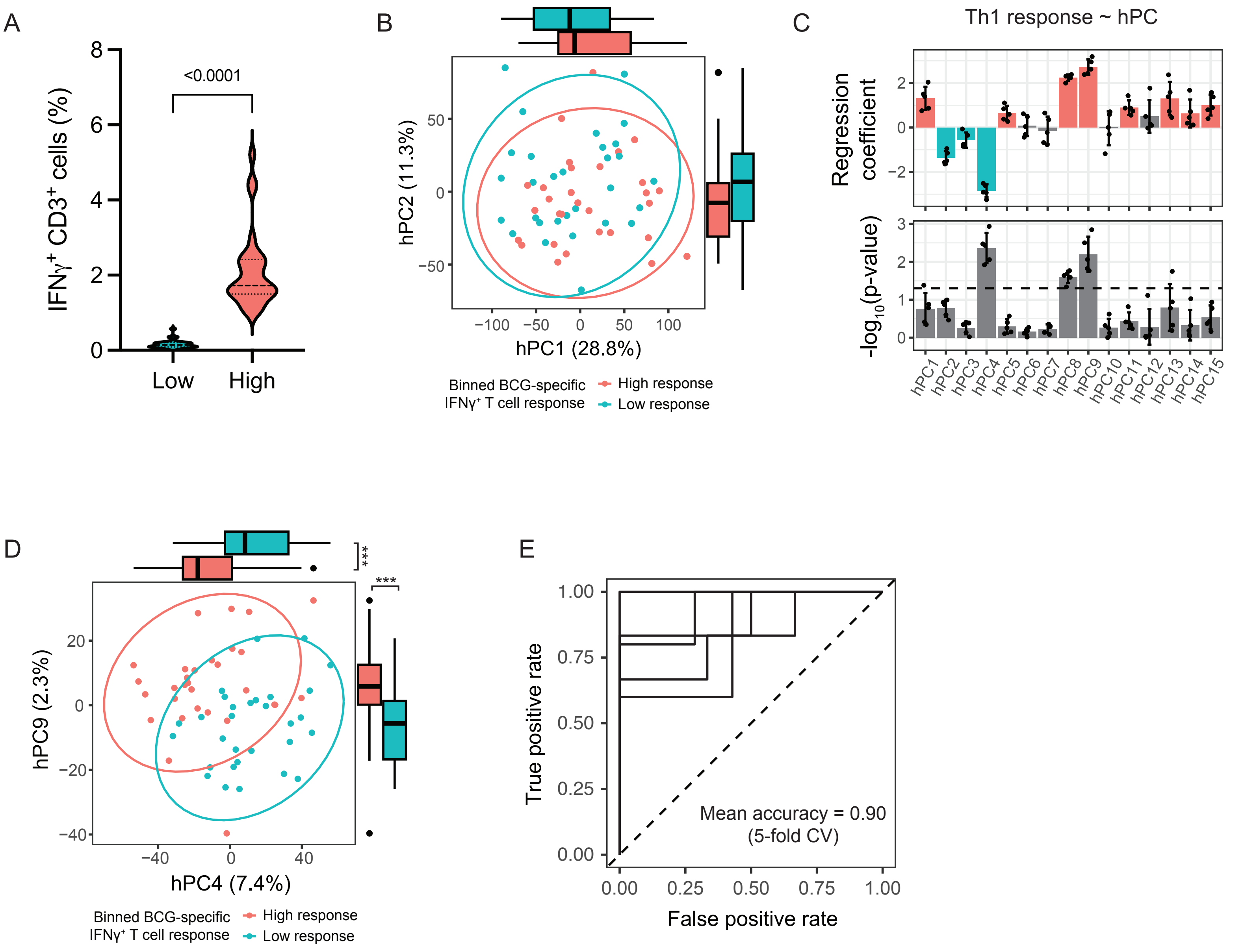

Related to Fig. 2.
(**A**) Percentage of CD3⁺ cells (T cells) expressing IFNγ upon *ex vivo* BCG stimulation separated into low (blue) and high (pink) responders. The dotted lines illustrate median and 1st and 3rd quartiles. P-value < 0.0001 by unpaired t-test as calculated in GraphPad.
(**B** and **D**) Human samples projected along (B) hPC1 and hPC2, and (D) hPC4 and hPC9. Each datapoint represents one sample, and the colored ellipses represent the 95% confidence interval for each phenotype group's distribution in each respective space. Boxplots on the x- and y-axes illustrate the distribution of the high (pink) and low (blue) BCG-specific T cell response samples on each axis respectively. Thick lines denote the median; hinges denotes 1st and 3rd quartiles; whiskers extend from hinges ± 1.5 * IQR; outliers are plotted individually. ***p<0.001 by Wilcoxon rank-sum testing.
(**C**) (top) Bar plot showing 5-fold logistic regression coefficients (summary data presented as mean ± SD) for each hPC when univariately regressed against human phenotype (BCG response). (bottom) Bar plot showing the distribution of corresponding p-values.
(**E**) ROC curves for 5-fold logisitic regression of human phenotypes using projected human data onto hPC4, hPC8, and hPC9 together.

Supplemental Figure 3: Interpretation of hPC-projected NHP data w.r.t. BCG vaccine dose

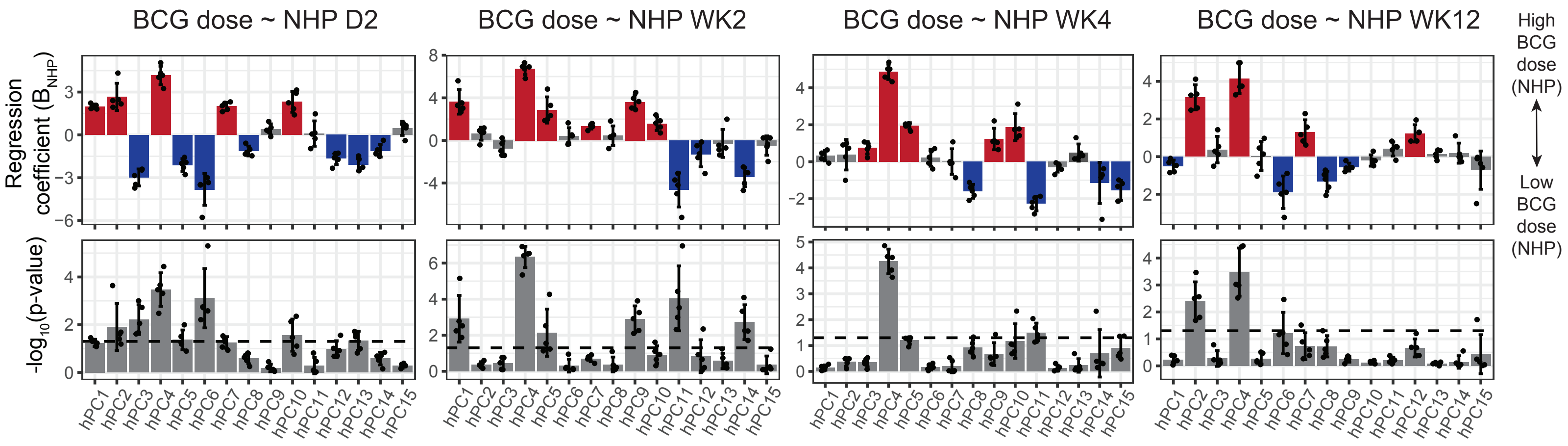

Related to Fig. 3.
Bar graphs showing (top) 5-fold logistic regression coefficients (summary data presented as mean ± SD) and (bottom) distribution of corresponding p-values from univariate regression of each hPC against BCG dose. The dotted line denotes p = 0.05.

Supplemental Figure 4: hPCs can reliably separate NHP samples by protection status, although model performance is dependent on time post-vaccination

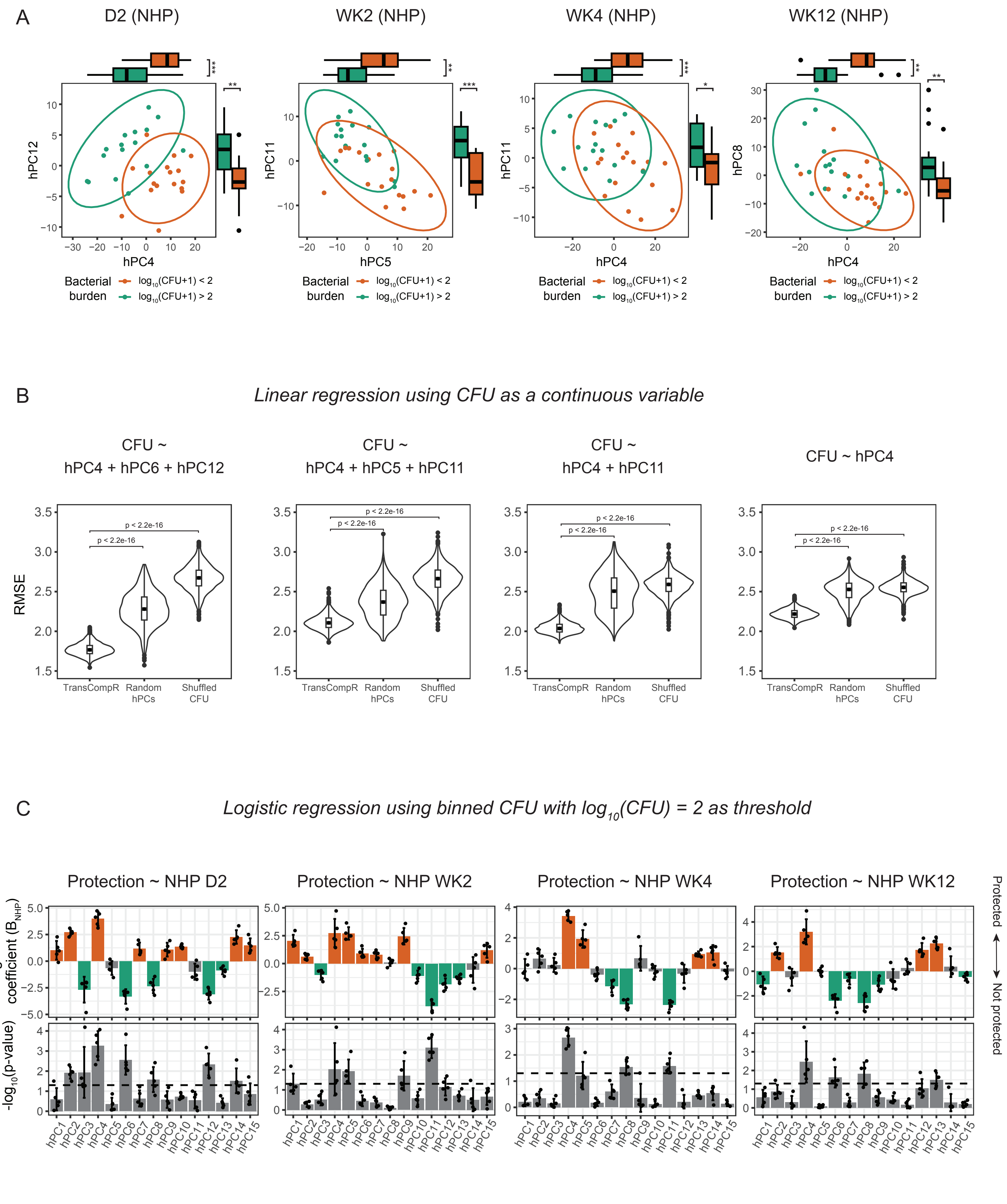

D

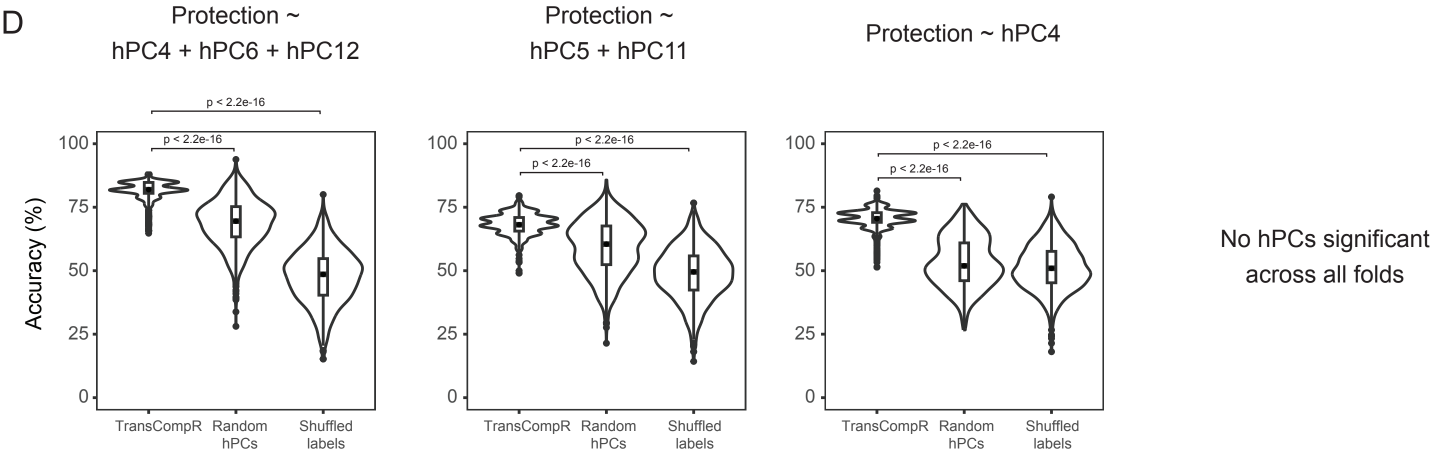

Related to Fig. 3.

(**A**) NHP samples from 4 post-vaccination timepoints projected along hPC4 and hPC12, hPC5 and hPC11, hPC4 and hPC11, and hPC4 and hPC8, respectively. Each datapoint represents one sample, and the colored ellipses represent the 95% confidence interval for each phenotype group's distribution in each respective space. Boxplots on the x- and y-axes illustrate the distribution of the high (green) and low (orange) CFU samples on each axis respectively. Thick lines denote the median; hinges denotes 1st and 3rd quartiles; whiskers extend from hinges ± 1.5 * IQR; outliers are plotted individually. *$p<0.05$, **$p<0.01$, ***$p<0.001$ by Wilcoxon rank-sum testing.

(**B**) Violin plots summarizing the test root mean square error (RMSE) for 1000 trials of 5-fold CV for covariate linear regression models constructed using (left) all hPCs that were statistically significant via univariate linear regression against numerical CFU (center) randomly selected hPCs, or (right) statistically significant hPCs and regressed against shuffled CFU. Boxplots within the violins are rendered as in (A). $p < 2.2e-16$ by Wilcoxon rank-sum testing.

(**C**) Bar graphs showing (top) 5-fold logistic regression coefficients (summary data presented as mean ± SD) and (bottom) distribution of corresponding p-values from univariate regression of each hPC against binned CFU (protected vs. not protected). The dotted line denotes $p = 0.05$.

(**D**) Violin plots summarizing the test accuracy (%) for 1000 trials of 5-fold CV for covariate logistic regression models constructed using (left) all hPCs that were statistically significant via univariate logistic regression against binned CFU in (C), (center) randomly selected hPCs, or (right) statistically significant hPCs and regressed against shuffled labels. Boxplots within the violins are rendered as in (A-B).$p < 2.2e-16$ by Wilcoxon rank-sum testing.

Supplemental Figure 5: Human-to-NHP translation with route cohort further implicates two protection-associated hPCs

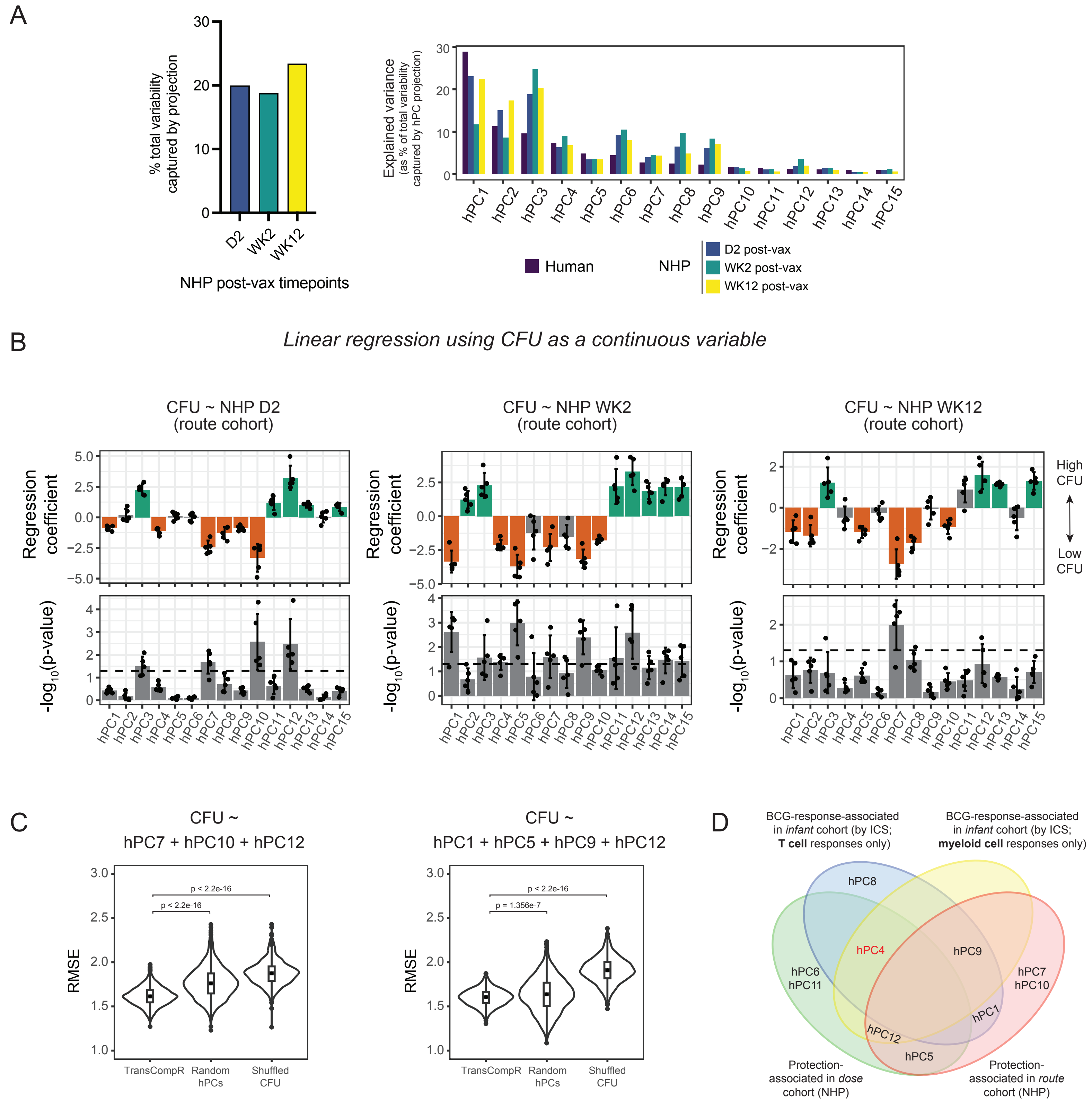

Related to Fig. 3.

(**A**) Bar graphs showing explained variance captured by projecting the NHP route cohort (GSE218157) into hPC space (left) as a percentage of total variance in each post-vaccination NHP dataset and (right) as distributed across each hPC in the TransCompR model.

(**B**) Bar graphs showing (top) 5-fold logistic regression coefficients (summary data presented as mean ± SD) and (bottom) distribution of corresponding p-values from univariate regression of each hPC against post-*Mtb* challenge bacterial burden (CFU). The dotted line denotes p = 0.05.

(**C**) Violin plots summarizing the test root mean square error (RMSE) for 1000 trials of 5-fold CV for covariate linear regression models constructed using (left) all hPCs that were statistically significant via univariate linear regression against numerical CFU in (B), (center) randomly selected hPCs, or (right) statistically significant hPCs and regressed against shuffled CFU. For boxplots within the violins: thick lines denote the median; hinges denotes 1st and 3rd quartiles; whiskers extend from hinges ± 1.5 * IQR; outliers are plotted individually. $p < 2.2e-16$ by Wilcoxon rank-sum testing.
(**D**) Four-way Venn diagram as in Fig. 3C which further separates BCG-specific immune cytokine responses in the South African infant cohort (by ICS) into T cell- and myeloid-specific designations.

Supplemental Figure 6: GSEA using KEGG database as reference provides additional support for biological interpretation of protection-associated hPCs

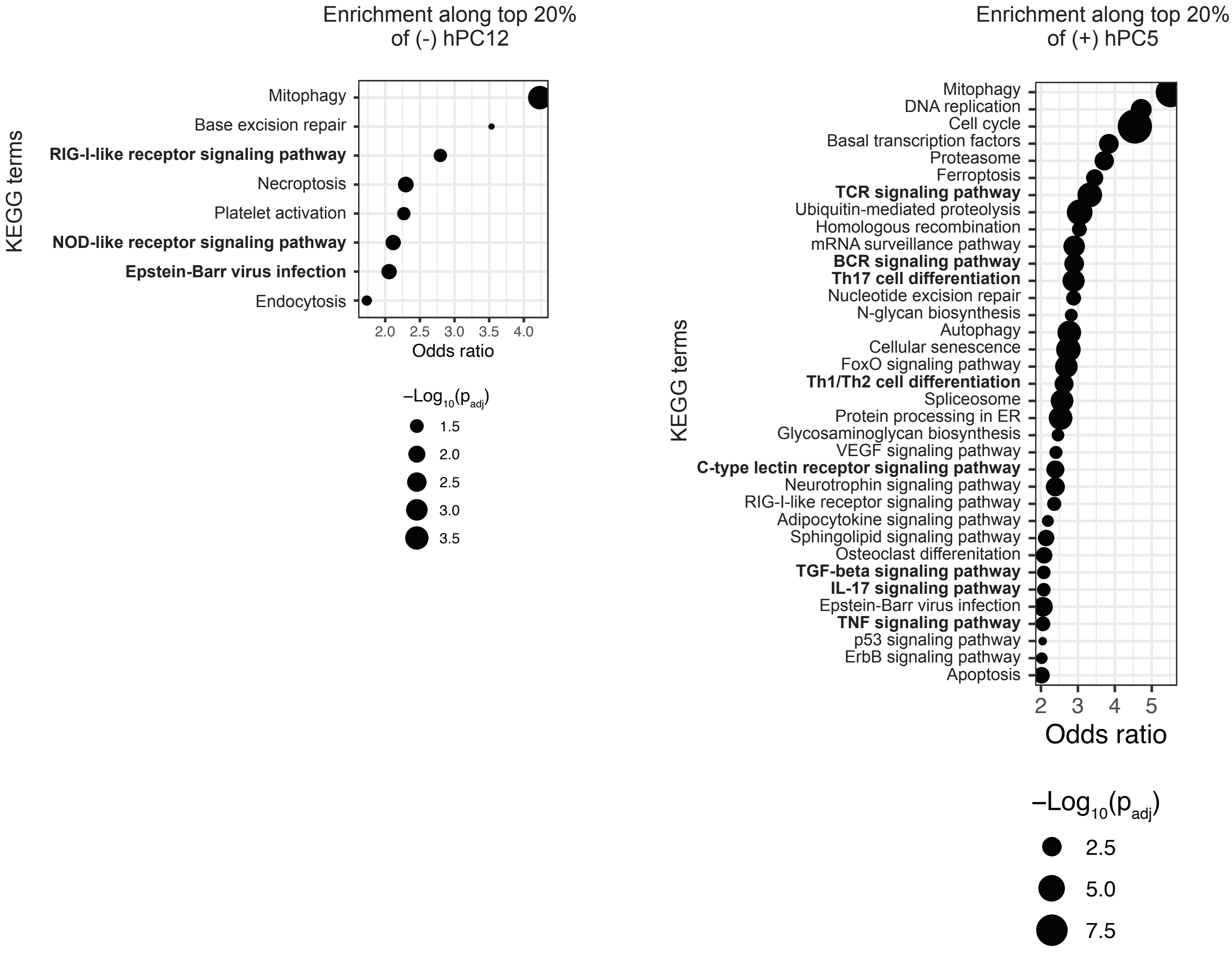

Related to Fig. 4.
KEGG terms significantly enriched on the ‘low CFU’-associated ends of hPC12 (left) and hPC5 (right). Dot size corresponds to adjusted p-value, and terms are plotted according to their odds ratio, which is a statistic that estimates the magnitude of the enrichment.

Supplemental Figure 7: ssGSEA on NHP route cohort confirms timepoint-dependent separation of samples by protection status along pathway-associated gene sets of interest

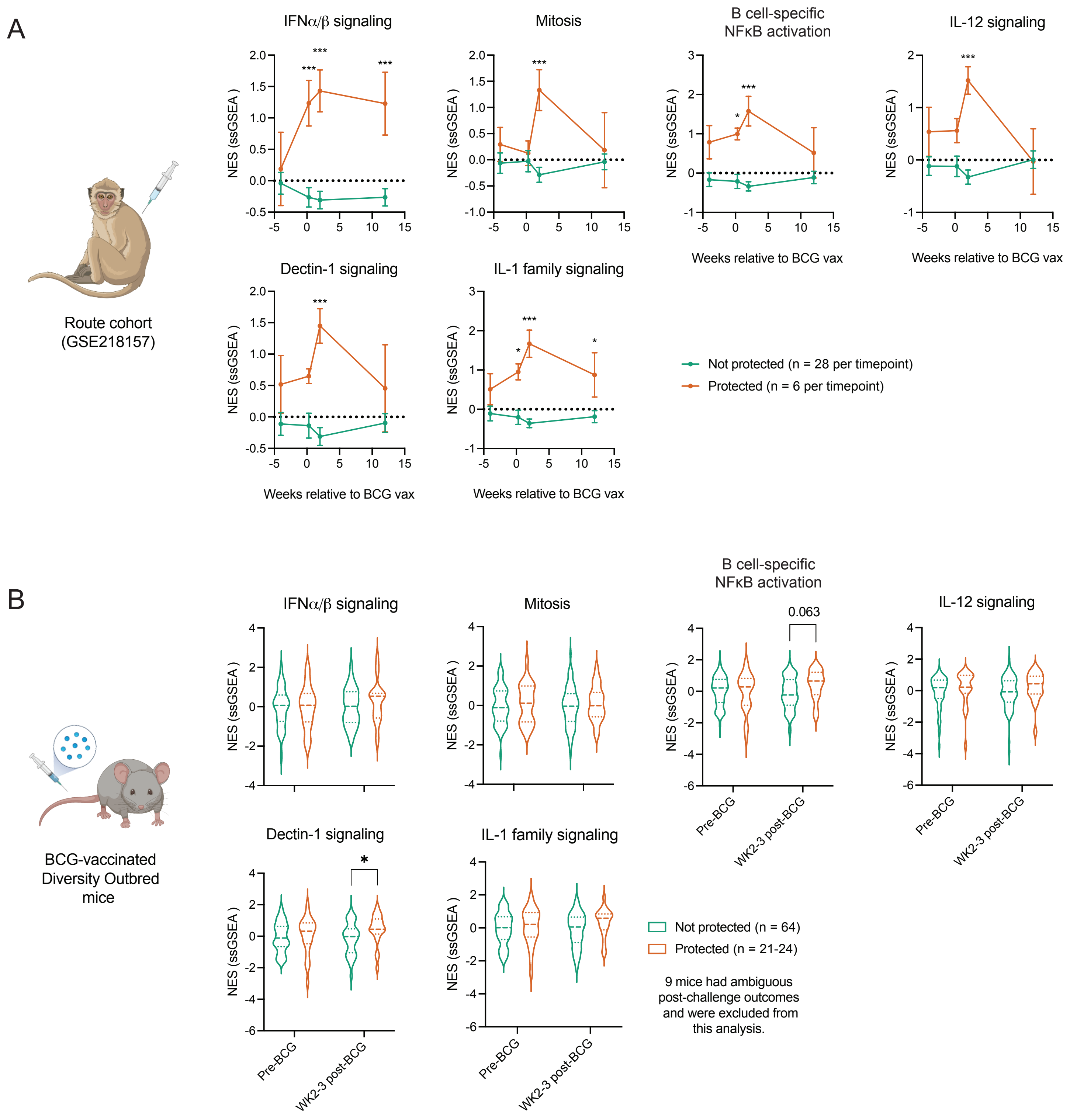

Related to Fig. 4.
Normalized enrichment scores (NES) calculated using single sample gene set enrichment analysis (ssGSEA) for each sample in the (**A**) NHP route cohort (GSE218157) and (**B**) a cohort of Diversity Outbred mice whose blood was collected 2-3 weeks before and after BCG vaccination for bulk RNA-seq. Gene sets for each pathway/process were derived from the Reactome Pathway Database. Sample scores were then lumped by protection outcome as evaluated post-*Mtb* challenge for visualization. *p < 0.05, **p < 0.01, ***p < 0.001 by unpaired t-test with Holm-Šídák's multiple comparison correction as calculated in GraphPad.

Supplemental Figure 8: GSE20716 provides partial validation for associations between hPCs and protection

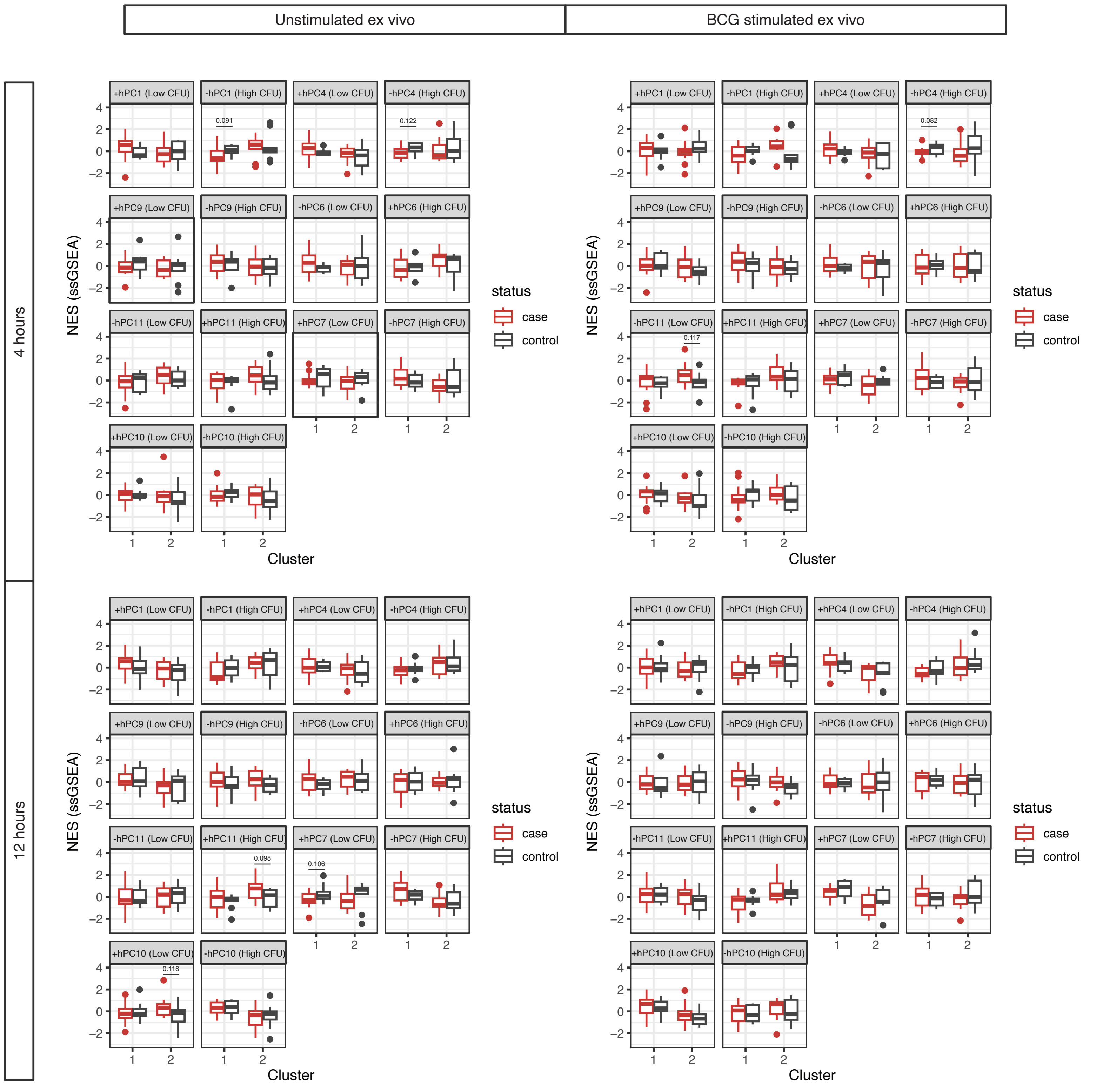

Related to Fig. 5.
Boxplots visualizing normalized enrichment scores (NES) calculated with ssGSEA for each microarray sample either (left) left unstimulated or (right) BCG-stimulated ex vivo for (top) 4 or (bottom) 12 hours. The thick center line of the box denotes the median; the bounds of the boxes (hinges) denote the 25th and 75th percentiles; the upper and lower whiskers extend ± 1.5 * interquartile range from the upper and lower hinges, respectively; data beyond the whiskers are considered outliers and plotted individually. Samples were separated by case (red) versus control (blue) follow-up outcome and by major cluster assigned in the original publication of this dataset. Statistical testing between distributions of enrichment scores between cases and controls was conducted by unpaired t-test.

Supplemental Table 1. Breakdown of 60 human samples by biological sex and binned BCG-specific $CD3^+IFN\gamma^+$ response assessed by ICS.

		BCG-specific $CD3^+IFN\gamma^+$ response (binned)		
		High	Low	Total
Biological sex	M	15	14	29
	F	15	16	31
	Total	30	30	60

Supplemental Table 2. Breakdown of 34 publicly available NHP samples by biological sex, administered binned BCG dose, and binned post-*Mtb* challenge lung bacterial burden (‘protected’ = $\log_{10}$(CFU) < 2; ‘not protected’ = $\log_{10}$(CFU) > 2).

**Post-*Mtb* challenge**	Protected (n=18)				Not protected (n=16)			
**BCG dose**	High (n=12)		Low (n=6)		High (n=4)		Low (n=12)	
**Sex**	M (n=5)	F (n=7)	M (n=2)	F (n=4)	M (n=1)	F (n=4)	M (n=8)	F (n=4)

Supplemental Table 4. Breakdown of 46 patients profiled in GSE20716 by two-year follow-up outcome and by assigned cluster from original publication.

		Cluster (original publication)			
		1	2	N/A	Total
**Follow-up outcome**	Case	15	11	0	26
	Control	9	9	2	20
	Total	24	20	2	46

Supplemental Table 5. *P* values calculated for case versus control comparisons presented in Figure 5 (Cluster 1 only).

	Unstimulated (4hr)	Unstimulated (12hr)	BCG (4hr)	BCG (12hr)
**(+) PC5**	0.3046187931	0.7508027765	0.1318235764	0.4902793332
**(-) PC5**	0.7052119076	0.8534844929	0.7180778071	0.8020508524
**(-) PC12**	0.5803166594	0.4923553891	0.9664058423	0.80576733
**(+) PC12**	0.06420062947	0.08551509112	0.3088395471	0.1049309701

Supplemental Table 6. Fisher's Method-merged *P* values for case versus control comparisons (Fig. 5, Supp. Fig. 8) using genes for all hPCs which were associated with protection in at least one cohort of NHPs.

	Cluster 1		Cluster 2	
	X^2	*P* value	X^2	*P* value
**hPC5 (+)**	8.4288	0.3927	4.0492	0.8527
**hPC5 (-)**	2.1189	0.9771	0.75852	0.9994
**hPC12 (-)**	3.0057	0.934	3.4525	0.9028
**hPC12 (+)**	17.268	0.02743	3.9969	0.8574
**hPC1 (+)**	5.7116	0.6795	2.7585	0.9486
**hPC1 (-)**	10.686	0.2202	5.2351	0.7322
**hPC4 (+)**	6.8073	0.5576	2.4604	0.9636
**hPC4 (-)**	14.248	0.07553	5.5995	0.692
**hPC9 (+)**	4.3848	0.8208	3.3805	0.9083
**hPC9 (-)**	2.4023	0.9661	7.9032	0.443
**hPC6(-)**	9.4305	0.3073	3.3207	0.9127
**hPC6 (+)**	1.7777	0.9871	3.4266	0.9048
**hPC11 (-)**	2.4949	0.962	7.8212	0.4511
**hPC11 (+)**	3.2103	0.9205	10.776	0.2147
**hPC7 (+)**	9.1406	0.3306	3.877	0.868
**hPC7 (-)**	8.946	0.3469	7.1433	0.5212
**hPC10 (+)**	1.8668	0.9848	8.4882	0.3873
**hPC10 (-)**	3.4668	0.9018	4.7004	0.7891
